# Identification and validation of reference genes for quantitative RT-PCR normalization in wheat

**DOI:** 10.1186/1471-2199-10-11

**Published:** 2009-02-20

**Authors:** Anna R Paolacci, Oronzo A Tanzarella, Enrico Porceddu, Mario Ciaffi

**Affiliations:** 1 Dipartimento di Agrobiologia ed Agrochimica, Università della Tuscia, Via S. Camillo de Lellis, 01100 Viterbo, Italy

## Abstract

**Background:**

Usually the reference genes used in gene expression analysis have been chosen for their known or suspected housekeeping roles, however the variation observed in most of them hinders their effective use. The assessed lack of validated reference genes emphasizes the importance of a systematic study for their identification. For selecting candidate reference genes we have developed a simple *in silico *method based on the data publicly available in the wheat databases Unigene and TIGR.

**Results:**

The expression stability of 32 genes was assessed by qRT-PCR using a set of cDNAs from 24 different plant samples, which included different tissues, developmental stages and temperature stresses. The selected sequences included 12 well-known HKGs representing different functional classes and 20 genes novel with reference to the normalization issue. The expression stability of the 32 candidate genes was tested by the computer programs geNorm and NormFinder using five different data-sets. Some discrepancies were detected in the ranking of the candidate reference genes, but there was substantial agreement between the groups of genes with the most and least stable expression. Three new identified reference genes appear more effective than the well-known and frequently used HKGs to normalize gene expression in wheat. Finally, the expression study of a gene encoding a PDI-like protein showed that its correct evaluation relies on the adoption of suitable normalization genes and can be negatively affected by the use of traditional HKGs with unstable expression, such as actin and α-tubulin.

**Conclusion:**

The present research represents the first wide screening aimed to the identification of reference genes and of the corresponding primer pairs specifically designed for gene expression studies in wheat, in particular for qRT-PCR analyses. Several of the new identified reference genes outperformed the traditional HKGs in terms of expression stability under all the tested conditions. The new reference genes will enable more accurate normalization and quantification of gene expression in wheat and will be helpful for designing primer pairs targeting orthologous genes in other plant species.

## Background

Transcriptome and gene expression analyses are contributing to substantially improve our understanding of the signalling and metabolic pathways underlying developmental and cellular processes. Quantitative RT-PCR (qRT-PCR) is currently one of the most powerful and sensitive techniques for analyzing gene expression and, among its several applications, it is often used for validating output data produced by micro- and macro-arrays of whole-genomes and as a primary source for detecting specific gene expression patterns [1-3]. Reliable quantification by qRT-PCR analysis of gene expression levels requires the standardization and fine-tuning of several parameters, such as: amount of initial sample, RNA recovery and integrity, enzymatic efficiencies of cDNA synthesis and PCR amplification, overall transcriptional activity of the tissues or cells analyzed [4,5]. Among several proposed methods [5,6], internal control genes (reference genes) are most commonly used to normalize qRT-PCR and to reduce possible errors generated in the quantification of gene expression, which is obtained by comparing the expression levels in the analysed samples of the gene of interest and of stable constitutive control genes. Obviously, the success of this procedure relies on the choice of appropriate control genes, which ideally would be those showing stable expression under various experimental conditions and in different tissues types. Housekeeping genes (HKGs), whose protein products are involved in basic cellular processes and are supposed to have stable and uniform expression across different tissues and developmental stages, are commonly exploited as internal controls for normalization in gene expression analyses. The reference genes most widely used for qRT-PCR in different species are those encoding 18S rRNA, actin, tubulin, polyubiquitin and GAPDH, which have commonly been employed in traditional methods of gene expression detection, such as northern blot, RNase protection test and conventional semi-quantitative RT-PCR [6]. However, several reports have shown that frequently the most widely used HKGs are not reliable controls, since their expression level varies in different tissues [7-13], and it would be essential a preliminary evaluation for identifying the most stable HKGs in each species. It is rapidly increasing the number of articles on the identification and validation of novel and more stable reference genes in mammalians, as well as the programmes of statistical software for evaluating the stability of selected HKGs [13-15]. On the contrary, there are few specific studies on expression analysis of HKGs in plants, most of them evaluating the expression in different species and experimental conditions of the same well-known reference genes [16-20]. Recently a novel set of reference genes has been identified in *Arabidopsis *(using the large public collection of data from Affymetrix GeneChip experiments) [12], and in barley (on the basis of ESTs analysis and relative expression calculation for all the cDNA libraries available through the TIGR Barley Gene index database) [21]. These studies indicate that many new reference genes outperforming the traditional ones in terms of expression stability can be found by different approaches. However, the increased awareness of the importance of systematic validation has not permeated fully throughout the community of plant molecular biologists, and although the potentially highly misleading effects of using inappropriate references for normalization are widely known, they are also widely disregarded. Gutierrez et al. [22] have shown that genes validated as references have been used in only 3.2% of 188 real-time RT-PCR analyses published during a 6-month period from July through December 2007 in the three leading primary research journals in plant biology, according to the ISI Web of Knowledge (The Plant Cell, Plant Physiology, and The Plant Journal). In another recent paper Gutierrez et al. [23] have also shown that genes commonly used as references may be expressed unstably during the development of *Arabidopsis *plants. Using validated and non-validated reference genes, the authors illustrated to what extent the use of arbitrarily chosen genes could affect the quantification of target gene expression levels. Up to 100-fold variations were found in the expression of a target gene, which could be attributed only to variations in the expression of the reference genes, with consequently huge potential scope for misinterpretation of the results. Therefore, there is an urgent need to regard the systematic validation of reference genes as an essential component of real-time RT-PCR analysis to improve the reliability of published results and retain the accuracy of this powerful technique.

Wheat is the most widely grown crop in the world and has paramount importance for human nutrition. Cultivated bread wheat (*Triticum aestivum *L.) is an allohexaploid species (AABBDD) with three very large homoeologous genomes, each comprising seven pairs of chromosomes. The whole hexaploid wheat genome is about 40 times larger than that of rice, this huge difference is largely due to the inclusion of repetitive sequences [24]. The close synteny between the rice and wheat genomes shown by genetic maps based on cDNA markers [25,26] indicates that their physiological and developmental differences are mostly due to regulation and expression patterns of similar structural genes. Since the wheat genome is too large to be entirely sequenced in the next future, structural and functional analysis of the wheat transcriptome is particularly important. The screening of the whole wheat transcriptome is made possible by the recent availability of an Affymetrix GeneChip Wheat Genome Array containing 61,127 probe sets (55,052 transcripts), likely covering half of the wheat expressed genes [27-29], and of several application-specific cDNA arrays [30-32]. Since qRT-PCR is necessary for array validation and accurate expression studies, it is important to identify reliable internal control genes suitable for many experimental conditions.

Using the terms "wheat", "gene expression", and "real time RT-PCR" linked by the Boolean operator "AND", we performed a PubMed search of articles published from January 1996 to March 2008 and retrieved 26 articles that used 16 different reference genes (Additional file 1). Remarkably, genes encoding 18S rRNA (8 times, 30%), actin (7 times, 27%) and alpha-tubulin (5 times, 19%) have been used in about 3/4 of the studies, whereas each of the remaining reference genes has been cited only 1–3 times (4–11% of the papers). Only four of the studies were based on multiple reference genes, whereas 22 researches used single reference genes, and 15 of them presumed their expression stability without any preliminary validation. Although 18S rRNA has been frequently used as a reference gene, it is far from ideal for several reasons: i) need of using total RNA and random primers for the RT reaction; ii) very high expression levels compared to the mRNA transcripts of the gene of interest; iii) imbalances in rRNA and mRNA fractions between different samples [33,34]; iv) expression affected by tissue and experimental conditions [4,33]; v) reduced degradation compared to mRNA [16]. Also the constitutive genes actin and alpha-tubulin, which are commonly used as reference genes in plants, do not represent the best choice because they are present in multiple copies showing high variation in their expression levels [35,36]. Our literature survey evidenced the lack of validated reference genes for expression studies in wheat and clearly emphasized the value of the present systematic study for identifying more reliable genes.

Microarray datasets can be a rich source of information for selecting qRT-PCR reference genes, as shown in *Arabdopsis *[12]; the same approach can not be followed in wheat for the limited availability of public microarray datasets [27-32]. As transcript abundance can be inferred by EST frequency, the computational analysis of ESTs from various cDNA libraries represents an alternative strategy. As of January 2008, about 1,000,000 wheat ESTs accumulated in the dbEST (NCBI) are freely available and represent an opportunity for extensive investigation to identify reference genes with stable expression across a wide range of developmental and environmental conditions. Wheat EST database contains sequences of cDNA libraries obtained from several tissues, including cell cultures, crown, spikes at different stage of development, flower organs (lemma, palea, anthers and pistils), seedlings, leaves, roots, stems, sheath, seeds at different stages of development, endosperm and embryo. Moreover, the same database includes EST sequences from cDNA libraries of wheat plants exposed to abiotic (salt, drought, high and low temperatures, aluminium) and biotic (*Fusarium graminearum*, powdery mildew, stripe rust, Hessian fly) stresses. The value of this resource is further enhanced by the availability of two public wheat-specific databases, *Triticum aestivum *UniGene and TIGR wheat Gene index, in which a set of non-redundant EST-index is produced. UniGene (NCBI) [37] is a system for partitioning GenBank sequences, including ESTs, into a non-redundant set of orientated clusters. Each UniGene cluster contains sequences representing a unique gene, which is linked to the tissue types where it is expressed and to characterized proteins of model organisms encoded by similar sequences. The over 947,303 wheat ESTs contained in GeneBank were reduced about 22-fold to 41,256 sequence clusters in *T. aestivum *UniGene April 2008 release (build 52). However, NCBI does not generate contigs and/or consensus sequences for UniGene clusters, whereas TIGR produces Gene Indices which are constructed by first clustering and then assembling ESTs sequenced at TIGR, ESTs from dbEST and annotated gene sequences from GenBank [38]. Each TIGR cluster contains a fasta formatted consensus sequence with a unique accession number (TC, Tentative Consensus), as well as additional information including details of the assembly, cDNA libraries from which the single assembled ESTs are derived and putative gene identification. The last release of *T. aestivum *Gene Index (release 11, July 13, 2008) contains 216,452 unique sequences, including 91,464 assembled TC sequences, 124,732 singleton ESTs and 256 singleton ETs (annotated sequences).

In the present research several novel candidate reference genes suitable for gene expression normalization in wheat were identified by a cross search for stable expression in Unigene and TIGR databases. The expression of 32 genes representing different functional classes was assessed by qRT-PCR of RNAs from 18 wheat tissues and floral organs and from seedlings exposed to low and high temperatures. Additionally, we carried out a comprehensive evaluation of the expression patterns of the actin and alpha-tubulin gene families, whose gene members have commonly been used as controls for the normalization of gene expression in wheat. The expression stability of the analysed genes was estimated by three statistical approaches: 1) threshold cycle (C_t_) variation range and coefficient of variation; 2) geNorm and 3) Norm Finder programs. Finally, by studying the expression of a gene encoding PDI (Protein Disulfide Isomerase) in different tissues and developmental stages of wheat it was shown that the correct evaluation of its expression relies on the adoption of suitable normalization genes and can be negatively affected by the use of genes with unstable expression.

## Methods

### *In silico *identification of reference gene candidates

The *in silico *approach was applied to identify genes with stable expression levels among different wheat tissues and included two subsequent steps. First the UniGene EST ProfileViewer resource was used to analyse the expression of 41,256 sequence clusters of UniGene *T. aestivum *database (build 52, released 11 April 2008). The ProfileViewer estimates the expression pattern of each Unigene cluster by relating the number of clustered ESTs to the total ESTs obtained from the cDNA libraries of ten different tissues: callus, cell culture, crown, flower, inflorescence, leaf, root, seed, sheath and stem; the gene transcription profile of each cluster in each tissue is reported as a value representing the number of transcripts per million (TPM). Three criteria were adopted for selecting wheat candidate reference genes from UniGene: 1) each UniGene cluster would contain at least 60 single ESTs; 2) each UniGene cluster would be expressed in all ten tissues; 3) the difference between the highest and the lowest expression value (TPMs) for each UniGene cluster would not be below 40% in the six most representative tissues: flower, inflorescence, leaf, root, seed and stem. Using such criteria, 177 out of the 42,256 sequence clusters were selected and mean value (Mv), variance (V), standard deviation (SD) and coefficient of variation (CV) were calculated on their expression values (TPM) for the six most representative tissues. An independent search for members of the α-tubulin and actin gene families carried out in UniGene identified ten more clusters, five for each gene family.

The second step consisted in the identification of the TC sequence corresponding to each of the 187 (177+10) selected UniGene clusters; the longest sequence, including part or the whole coding region and the 3' UTR, was identified, downloaded and exploited to BLAST search the TIGR wheat gene index database (TaGI version 11). This database is in many ways complementary to other gene indexing databases and its clustering and high stringency approach offer a number of significant advantages over alternatives such as UniGene. First, assembly provides a high confidence consensus representing each transcript, whereas low quality, misclustered or chimeric sequences are identified and discarded; moreover, closely related but distinct transcript isoforms can be recognized when sufficient sequence extent is available. Second, the produced consensus sequence is generally longer than its individual ESTs, providing a resource that can be used more effectively for functional annotation and for designing specific primers for its expression analysis. The TIGR Gene Indices compare and cluster ESTs and annotated cDNA sequences when they meet the following criteria: I) at least 40 base pair match; II) identity higher than 94% in the overlapping region; III) maximum 30 base pairs of unmatched overhang. These clusters are then assembled into a consensus sequence using Paracel Transcript Assembler [38]. UniGene applies different parameters: EST sequences are linked in a cluster if there is a 50 bp overlapping (100% identity) within the 3' UTR. However the identified clusters are not further checked through the more stringent criteria of assembly process and consensus sequences, this explains why sometimes several closely related but not matching TCs were included into the same UniGene cluster. Therefore we selected only the TCs showing identity higher than 95% with the longest sequence representative of each UniGene cluster. Since the more stringent assembly process used by TIGR enhances the detection of splicing variants, in the allohexaploid genome of *T. aestivum *it most likely allows the identification of closely related but distinct transcripts of homoeologous genes. For each TC or group of TCs linked to the 187 selected UniGene clusters, we determined their frequency (number of hosting libraries/total number of cDNA libraries) to find out the TCs reported in a significant number of different cDNA libraries and, consequently, more stable and suitable to be included in a preliminary extended set of candidate reference genes. Out of the 250 cDNA libraries used to construct the TIGR Wheat Gene Index (release 11), TC frequency counting was restricted to the libraries (197) containing at least 100 ESTs, because the remaining 53 include a limited number of clones deriving from differential display and cDNA-AFLP analyses or from SSH libraries. Functional classification of each UniGene cluster and linked TC or TCs was performed on the basis of its rice orthologous genes and relative GO (Gene Onthology) annotations.

### Plant material

The following tissue samples were collected (January-June 2007) from 20 bread wheat plants (*T. aestivum *cv Chinese Spring, CS) grown in open field at Viterbo (Italy), immediately frozen in liquid nitrogen and kept at -80°C until use: 1) roots from plants with single shoot and three leaves unfolded (Feekes scale 1.3); 2) the above-ground portion from the same plants; 3) shoots at the beginning of tillering (Feekes scale 2); 4) shoots from plants with formed tillers (Feekes scale 3); 5) shoots at the beginning of erect growth (Feekes scale 4); 6) stems at booting stage (Feekes scale 10); 7) flag leaves at booting stage (Feekes scale 10); 8) spikes collected at intervals of 10–12 days (three developmental stages: 15–20 mm, flag leaf unfolding and heading stage); 9) single floral organs (glumes, palea, lemma, lodicules, stamens and pistil) from fully emerged spikes (Feekes scale 10.5); 10) caryopses collected 15 (medium milk stage) and 30 (hard dough stage) days after anthesis.

For temperature stress experiments seeds of *T. aestivum *cv CS were germinated on cotton pads 6 days at 18°C, 16 h light. Seedlings were transplanted into small pots with soil and grown for 20 days (until reaching the third leaf unfolding stage, Feekes scale 1.3) at 18°C with 16 h light at 65% relative humidity. White fluorescent and incandescent lighting was combined to provide a light intensity of 196 μmol m^-2 ^s^-1^. Temperature treatments were performed exposing pots to 33°C and 4°C for 48 h. Two biological replications (20 plants) were exposed to each treatment. The aerial parts of the plants were harvested just before the start of temperature treatments (control plants), after 24 h and 48 h of exposition to 33°C and 4°C, frozen in liquid nitrogen and kept at -80°C until use.

### RNA isolation and cDNA synthesis

From most samples total RNA was extracted using the TRIzol reagent (Invitrogen) according to manufacturer's instructions, but adding an additional chloroform extraction. Total RNA from caryopses was isolated by a LiCl based method [39]. The resulting RNA was treated with Rnase-free DNase I (Promega) according to the manufacturer's protocol. Following digestion, nucleotides were removed from RNA using a G50 sepharose buffer exchange column (Amersham). Absence of genomic DNA contamination in DNase I-treated samples was checked by qRT-PCR of 0.125 μg of RNA template using a primer pair (5'-CTTATGCAATCCAATGATGG-3' and 5'-TCTATGGTTCTGAAGAGGACC-3') designed to amplify an intron sequence of a gene encoding PDI and located in chromosome 4A [40]. When a single DNase treatment did not completely remove interfering genomic DNA, a second DNase incubation was performed to eliminate any detectable DNA. RNA concentration and integrity were checked with a UV/VIS spectrophotometer Lambda 3B (Perkin Elmer) before and after DNase I digestion. Only RNA samples with 260/280 wavelength ratio between 1.9 and 2.1 and 260/230 wavelength ratio greater than 2.0 before and after DNase I digestion were used for cDNA synthesis. The quality of RNA samples was also assessed by electrophoresis on 1% formaldehyde agarose gels. First-strand cDNA was synthesized from 3 μg of total RNA by Expand™ Reverse Transcriptase (RT) (Roche) and diluted 1:5 before use in qRT-PCR assays.

### RT-PCR analysis of cold- and heat-responsive genes

The effect of temperature stresses was checked by the expression analyses of the wheat genes *wcor14 *and *TaHSP101B*, which are specifically induced by cold and heat stresses, respectively [41-44]. Expression analyses were carried out by semi-quantitative RT-PCR because transcripts of both genes were not detected in plants grown at 18°C (control plants in our temperature experiments), but were immediately induced by heat (*TaHSP101B*) and cold (*wcor14*) stress treatments.

The gene *wcor14 *encodes an acidic and hydrophobic protein of about 140 aa with unknown function, with 70% identity with the barley chloroplast-imported COR14b polypeptide and a nearly identical N-terminal, putative chloroplast transit peptide of 51 amino acid residues [41]. Transcripts of *wcor14 *accumulated within 3–6 hours of cold acclimation at 4°C and reached a maximum after 3 days [41]. A search in the NCBI database found four sequences of *Triticum aestivum *encoding wcor14: wcor14a (accession number AF207545), wcor14b (accession number AF207546) [41], WCOR14a (Accession number AF491838) and WCOR14c (AF491837) [42]. The primers used for *wcor14 *RT-PCR analysis (forward: 5'-TGGGATGCCACCAAAGAC-3'; reverse: 5'-TGATACGCAAATGTTGAGC-3'), which were designed in conserved regions of the four cDNA sequences identified in the NCBI database, amplified a product of 94 bp.

Proteins of the HSP100 family are involved in the process of thermotolerance acquisition, in particular in renaturing proteins denatured by heat. They are ATPase involved in assembly/disassembly of protein complexes such as the ATP-dependent dissolution of cytosolic or nuclear protein aggregates forming during heat stress [45]. In wheat three gene sequences of the HSP100 family have been isolated and characterised: *TaHSP101A*, *TaHSP101B *and *TaHSP101C *[43]. To study the effect of high temperature treatment we chose the gene *TaHSP101B *because it showed the highest induction by heat shock [43,44]. A search in the NCBI database identified two sequences of *Triticum aestivum *encoding TaHSP101B: *HSP101 *(Accession number AF083344) [46] and *HSP101B *(Accession number AF097363) [43]. The same search found also two additional sequences isolated from *T. durum *(tetraploid wheat) and encoding TaHSP101B: *TdHSP101B-A *(Accession number AJ970533) and *TdHSP101B-B *(AJ970534), corresponding to homoeologous genes located in the long arm of chromosomes 1A and 1B, respectively [44]. The primers used for RT-PCR analysis of *TaHSP1001B *(forward: 5'-GACGCAGCTGTCCAAGAT-3'; reverse: 5'-GCACCTGGATGAGGATGT-3') were designed in conserved regions of the four cDNA sequences and amplified a product of 161 bp.

First-strand cDNA was synthesized as described above and the PCR reactions were performed by the TripleMaster PCR system (Eppendorf) using 1 μl of the RT reaction. After initial denaturation at 94°C for 2 min, amplification conditions were 35 cycles each at 94°C for 30 s, 60°C for 1 min and 72°C for 3 min, followed by a final extension step at 72°C for 7 min. RT-PCR of the constitutive actin gene (UniGene cluster Ta54825), one of the most stable genes in temperature treatments (see below), was performed as control. Samples of the amplification products (3 μl) were collected after 28, 32 and 35 PCR cycles and analysed by electrophoresis on 2% agarose gel. Each RT-PCR experiment was independently repeated twice to test amplification reproducibility. The specificity of the amplicons was checked by sequencing of the PCR products in order to confirm that its sequence corresponded to the target gene.

### Design and validation of qRT-PCR primers

Specific primer pairs were designed for 32 candidate reference genes and for five distinct members for each of the α-tubulin and actin gene families on the basis of their TC sequences. When multiple TC sequences linked to a single UniGene cluster were identified, the primers were designed in their conserved regions. Primer design and optimization were done by the Beacon Designer 6 software (STRATAGENE) imposing the following stringent criteria: T_M _of 55°C ± 2°C, PCR amplicon length between 60 and 280 bp, primer length of 20 ± 2 nt, and 40 to 60% guanine-cytosine content. Primers were also designed at the 3' end region of each TC sequence to encompass all potential splice variants and to ensure equal RT efficiencies. The complete set of primer pairs and their amplicon length, along with the corresponding UniGene clusters and linked TC sequences, are listed in Additional file 2. Single sequence amplifications were performed using gene-specific primer pairs, whereas multiple genes of the same family were also amplified by primer pairs designed in conserved regions [TEF-1α (m), ADP-RF (m), Actin (m) and α-tubulin (m)]; m representing amplification of multiple genes). Each of the primer pairs TEF-1α (m) and ADP-RF factor (m) amplified the transcripts of two identified homologous genes (Ta659/Ta53964 and Ta45379/Ta2291, respectively) within their gene families, whereas each of the primer pairs targeting conserved regions of the Actin (m) and α-tubulin (m) families amplified the transcripts of five genes. Preliminary qRT-PCR assays carried out on a pool of all available cDNAs using six different primer concentrations ranging from 50 to 300 nM showed that the optimal primer concentration was 150 nM, because it generated the lowest C_t _value, a sharp peak by melting curve analysis and absence of non-specific products or primer-dimer artefacts. The specificity of the amplicons was also checked by electrophoresis on 2% agarose gel and sequencing of the PCR products in order to confirm that the product sequence was the same as the target gene. Five-point standard curves of a 5-fold dilution series (1:1–1:625) from pooled cDNA were used for PCR efficiency calculation of each primer pair. The PCR efficiency (E) is given by the equation E = (10^[-1/m]^-1) × 100 [34], where m is the slope of linear regression model fitted over log-transformed data of the input cDNA concentration versus C_t _values according to the linear equation y = m*log(x) + b.

### qRT-PCR conditions

qRT-PCR analysis was performed using an Mx3000PTM real time PCR system with Brillant SYBR green QPCR master mix (STRATAGENE) according to manufacturer's protocols. qRT-PCR assays were carried out in 25 μl reaction volumes containg 1 μl of each diluted cDNA (1/5) and 150 nM forward and reverse primers. No template and RT-minus controls were run to detect contamination, dimer formation and presence of genomic DNA. As described previously, for each primer pair PCR efficiency was calculated in each run from a pool of all available cDNAs. The thermal profile comprised three segments: 1) 95°C for 10 min; 2) 40 cycles of 30 s denaturation at 95°C, 1 min annealing at 55°C and 30 s extension at 72°C (amplification data collected at the end of each extension step); 3) dissociation curve consisting of 1 min incubation at 95°C, 30 s incubation at 55°C, a ramp up to 95°C. Two biological replicates, resulting from two different RNA extractions, RT and qRT-PCR reactions, were used for quantification analysis and three technical replicates were analysed for each biological replicate. Gene expression levels were recorded as C_t _values, which are inversely related to the initial DNA concentration. The built in amplification-based proprietary algorithm (STRATAGENE) was used to set the fluorescence threshold value for each primer pair reaction. This algorithm determines the portion of the amplification plots where all data curves display an exponential increase in fluorescence and calculate the threshold value that minimizes the standard deviation in C_t _values for each replicate set at a point which falls within 5–60% of the fluorescence shift for all curves. The number of cycles (C_t_) at which the amplification-corrected normalized fluorescence (dRn) for each reaction crossed the threshold value was exported to Excel (Microsoft) for further analyses.

### Assessment of expression stability

The expression stability of the analysed genes was estimated by three different statistical approaches. The first one consisting of single factor ANOVA on raw C_t _values of each gene was performed using the Excel Analysis ToolPak; genes showing high variance between the analysed samples should be avoided as controls. In this approach the C_t _difference (C_t_max-C_t_min) and CV for each gene were also computed to allow for a straightforward assessment of the most suitable reference genes that had a narrow C_t _range over all tissues and stress conditions analysed.

In the second and third approaches the stabilities among the samples of the candidate reference genes were evaluated by the software programs geNorm, version 3.4 [13] (Visual Basic application tool for Microsoft Excel available on the Internet) and NormFinder [15] (a Microsoft Excel Add-in available on the Internet) according to the author's recommendations. For both programs raw C_t _values were transformed to relative quantities using the delta-C_t _formula Q = E^ΔCt^, where E is the efficiency of the primer pair used in the amplification of a particular gene and ΔC_t _is the difference between the sample with the lowest C_t _(highest expression) from the data set and the C_t _value of the sample in question.

The statistical algorithm GeNorm determines a measure of gene expression stability (M value) corresponding to the average pairwise variation of a single candidate reference gene to all other genes under investigation. The M-value measure relies on the principle that the expression ratio of two hypothetical ideal internal control genes is identical in all samples, regardless of the experimental conditions or treatments. In this way, variation of the expression ratios of two candidate reference genes reflects the fact that one (or both) of the genes is (are) not constantly expressed, with increasing variation in ratio corresponding to decreasing expression stability (M value). Genes with the lowest M values have the most stable expression and therefore would be selected as ideal reference genes. In iterative steps, genes with the lowest expression stability (i.e. the highest M value) are removed. A new M value for each of the remaining genes is calculated until only two genes remain. Because these calculations are based on ratios, the final 2 genes cannot be resolved.

NormFinder assesses the expression stability of a gene by evaluating its expression variation within tissues or treatments ("groups" in NormFinder terminology) compared to variation among tissues/treatments. On the basis of a given "group identifier" the program can in fact discriminate between different groups, e.g. stem, leaf, spike or seedliings exposed to low or high temperatures. The program algorithm implies the estimation of intra- and inter-group variation and combines both results in a stability value for each investigated gene. The candidate gene with the lowest stability value is the most stable gene within the groups studied. The best combination of two genes is also indicated. In our analyses each tissue and temperature treatment is considered an experimental group, so we had 24 groups (18 different tissues and 6 temperature treatments, including controls and 24 and 48 h at 4°C and 33°C), each composed of the mean values of the two biological replicates.

### Reference gene validation

To determine how the adoption of different reference genes can affect the normalization of the expression data for a gene of interest, the same 24 plant cDNA samples used for the stability analyses of reference genes (see Plant material) were also analysed by qRT-PCR for the expression of TaPDIL1-1 (a gene encoding the Protein Disulfide Isomerase). Isolation and characterization in wheat of the three homoeologous gene sequences encoding PDI (TaPDIL1-1) and of their full-length transcripts have been reported previously [40]. The primer pair used for TaPDIL1-1 qRT-PCR analysis (forward: 5'-CGTGGTCTTCAAATCCTG-3'; reverse 5'-GTAACCCTGGACATCAAAC-3') was designed in conserved regions of the three homoeologous cDNA sequences at their 3' ends (Accession numbers: AJ868105, AJ868106, AJ868107) with annealing temperature of 55°C. The PCR efficiency was 100.35 ± 0,365 with a coefficient of determination (R^2^) of 0,998; the amplicon size was 187 bp. The TaPDIL1-1 expressions were normalized using five different strategies: 1) geometric average of the three references genes selected as the most stable genes by geNorm; 2) geometric average of the two references genes selected as the most stable genes by NormFinder; 3) the single most stable gene identified by NormFinder; 4–5) the two genes related to the Unigene clusters Ta54825 (Actin) and Ta25534 (α-tubulin) used alone. Normalized TaPDIL1-1 relative values are given as mean value ± SD. Standard deviations on normalized expression levels were computed according to the geNorm user manual [47].

## Results

### Identification of reference gene candidates

As described in the section "Methods", the approach adopted for the identification of wheat reference candidate genes included two subsequent steps. First the expression of 41,256 sequence clusters of UniGene *T. aestivum *database was analysed using the EST ProfileViewer resource (Additional file 3 reports the 177 selected UniGene clusters sorted in ascending order on the basis of their TPM CVs). Since UniGene clusters can not generate contigs and/or consensus sequences, the second step consisted in the identification of TC or group of TCs corresponding to each cluster using the TIGR wheat gene index database (TaGI version 11), whose outputs are more effective for gene functional annotation and for designing qRT-PCR primers. On the basis of the frequency (number of hosting libraries/total number of cDNA libraries) of each TC or group of TCs linked to the 177 selected UniGene clusters it was possible to pinpoint the TCs which, being represented in a significant number of cDNA libraries from different tissues, were the most suitable candidate reference genes to include in a preliminary screening. Functional classification of single TCs or groups of TCs linked to UniGene clusters was performed on the basis of their homology to rice orthologous genes and relative GO (Gene Onthology) annotations (Additional file 4). Of the 177 initially selected TCs or groups of TCs, 34 (19.21%) were homologous to rice genes encoding putative proteins whose biological functions are either unknown or not clear (Table 1); based on their presumed biological functions (GO biological process annotations), the remaining 143 selected sequences related to characterized rice genes were assigned to ten functional categories (Table 1). The categories "Cellular organization" and "Metabolism" contained the highest number of genes (18.08% and 17.51%, respectively), followed by "Posttranscriptional modification" (15.82%), "Protein synthesis" (9.04%) and "Signal transduction" (7.34%). These five functional categories were also reported among the most represented in sets of candidate human housekeeping or maintenance genes selected through microarray analysis using different Affymetrix platforms [48-50]. The remaining five categories ("RNA processing", "Energy", "Transcription", "Biotic and abiotic stress related" and "Development") were under-represented in the 177 selected sequences, accounting for only 12.99% (Table 1). The GO terms relative to biological processes over-represented among the different functional categories were determined to gain insight on the functional roles of the 177 selected sequences (Table 1). They included genes mediating a variety of basic cellular functions, such as: protein transport, ubiquitin-dependent protein degradation, proteolysis, protein translation, primary metabolism (amino acid biosynthesis, glycolysis and tricarboxylic acid cycle). Moreover, two of the over-represented GO terms referred to genes involved in the regulation of transcription (transcription factors) and in the intracellular signalling cascade (protein kinases). On the basis of the number of EST sequences included in each UniGene cluster the selected sequences were also assigned to four classes according to their level of expression: 1) high (>800 ESTs, 8.47%); 2) moderate (<800/>300 ESTs, 18.08%); 3) low (<300/>100 ESTs, 47.46%); 4) very low (<100 ESTs, 25.99%) (Additional file 5).

**Table 1:** Funcional classification and Gene Ontology (GO) terms over-represented in biological process for the 177 selected wheat candidate reference genes.

	**Functional Class**	**Representations (%)**	**GO terms**	**Biological process**	**NS (GO)**	**(%FC)**	**(%TS)**
1)	Biological function not annotated	34 (19.21)					
2)	Cellular organization	32 (18.08)	GO:0015031	Protein transport	15	46.88	8.47
			GO:0006810	Transport	8	25.00	4.52
3)	Metabolism	31 (17.51)	GO:0006520	Amino acid metabolic process	7	22.58	3.95
			GO:0006007	Glucose catabolic process	6	19.35	3.39
			GO:0006099	Tricarboxylic acid cycle	5	16.12	2.82
4)	Posttranslational modification (protein turnover)	28 (15.82)	GO:0006508	Ubiquitin-dependent protein catabolic process	15	53.57	8.47
			GO:00016925	Proteolysis	8	28.57	4.52
5)	Protein synthesis	16 (9.04)	GO:0006413	Translation initiation	8	50.00	4.52
			GO:0042254	Ribosome biogenesis and assembly	6	37.50	3.39
6)	Signal transduction	13 (7.34)	GO:0007243	Protein kinase cascade	5	38.46	2.82
7)	RNA processing	7 (3.96)					
8)	Energy	6 (3.39)	GO:0022900	Electron transport	6	100	3.39
9)	Transcription	5 (2.83)	GO:0045449	Regulation of transcription	5	100	2.82
10)	Biotic and abiotic stress related	3 (1.69)					
11)	Development	2 (1.13)					

NS = Number of sequences assigned to a GO term for biological process

%FC = Percentage of sequences assigned to a GO term for biological process relative to a particular functional class

%TS = Percentage of sequences assigned to a GO term for biological process relative to the total (177) selected sequences.

Most sequences identified as reference gene candidates were new, but they included also most of the known HKGs used as internal controls in gene expression analyses, such as GAPDH (Ta30768), malate dehydrogenase (Ta53937), β-tubulin (Ta44405), histone (Ta38797), cyclophylin (Ta35497), ribosomal proteins (Ta55103, Ta27771, Ta55901, Ta54208, Ta55238, Ta54141), members of the translation initiation and elongation factors gene families (Ta15761, Ta746, Ta14227, Ta1092, Ta54575, Ta53964, Ta659), ubiquitins (Ta50503, Ta55221), ubiquitin-conjugating enzymes (Ta56067, Ta54238, Ta24654) and proteasome subunits (Ta55216, Ta21374, Ta20028, Ta22845, Ta55217, Ta54768, Ta14021) (Additional file 4). Surprisingly, none of the genes belonging to the families encoding actin and α-tubulin met the three criteria adopted for selecting wheat reference candidate genes from UniGene. Since these genes have commonly been used as controls for normalization of gene expression in wheat, we decided to perform a detailed search for members of these two gene families in UniGene simply using the terms "actin" or "alpha-tubulin", and "wheat" combined by the Boolean operator "AND". Out of the 35 and 13 UniGene clusters identified for actin and α-tubulin, respectively, using the UniGene EST ProfileViewer resource we selected those containing at least 60 single ESTs and whose expression was detected at least in the six most representative wheat tissues: flower, inflorescence, leaf, root, seed and stem. Adopting such criteria five distinct UniGene clusters were identified for each of the two gene families and the Mv, V, SD and CV were calculated on their expression values (TPMs) for the six most representative tissues (Additional file 6). The CV values of three members of actin and five of α-tubulin gene families were higher than those of all the 177 UniGene clusters selected on the basis of the three stringent criteria adopted for selecting wheat reference candidate genes (compare Additional files 3 and 6), whereas the two lowest CV values (37.25 and 38.38) obtained for the two actin UniGene clusters Ta54825 and Ta53908 performed better than those of only 18 and 14, respectively, of the 177 selected sequences. Additionally, in all the members of actin and α-tubulin gene families the difference between the highest and lowest expression value (TPM) detected in the six most representative wheat tissues was inferior to 40% (Additional file 6). However, we decided to include all the 10 sequences in the qRT-PCR analysis to select the most stable member for each gene family and to compare them with all the other candidates selected as reference genes. For each UniGene cluster we identified the corresponding TC or group of TCs and determined their frequency (number of hosting libraries/total number of cDNA libraries). The five UniGene clusters and related TCs identified for the α-tubulin corresponded to the five non-homoeologous gene family members recently cloned in wheat [36].

### Selection of the best candidate reference genes for qRT-PCR analyses

Table 2 lists the 32 UniGene clusters and corresponding TCs selected for the analysis of their expression by qRT-PCR of RNA from 18 different wheat tissues and developmental stages and from seedlings exposed to low and high temperatures. These gene sequences were chosen considering three main criteria: 1) the ranking of the 177 reference gene candidates on the basis of the CV of their expression values (TPMs) in six wheat representative tissues (UniGene Profile Viewer); 2) the ranking of the TCs or groups of TCs linked to each of 177 UniGene clusters on the basis of their frequency (number of hosting libraries/total number of cDNA libraries); 3) their level of expression estimated as the number of EST sequences comprised in each UniGene cluster. Because one aim of the present research was to compare the effectiveness of traditional and novel reference genes, we included some of the most common HKGs. Finally, special attention was paid in order to select genes involved in distinct biological processes, this would theoretically reduce the chance of including co-regulated genes. However, for the purpose of testing the expression stability of sequences belonging to the same gene family, we also included multiple genes encoding ADP-ribosylation factors and elongation factors with very different expression levels (Table 2). Out of the 32 sequences selected as potential reference genes for wheat, 12 were related to well-known HKGs representing different functional classes, whereas the remaining 20 (in italics in Table 2) included genes that, according to literature searches, were not related to any housekeeping activity or genes with unknown functions, hence they can be considered novel with respect to the normalization issue. The first 11 Unigene clusters and corresponding TCs reported in Table 2 were selected because they ranked among the top 25 sequences with the lowest CV for their expression values (TPM), whereas 16 additional clusters (12–27 in Table 2) were chosen because they ranked among the 25 top sequences with the highest frequencies. Three more UniGene clusters (28–30 in Table 2) were selected for their known or suspected housekeeping roles in basic cellular processes. Eventually, the UniGene cluster Ta55512 (31 in Table 2) was included because it was related to a cathepsin B-like cysteine protease, the only chosen gene assigned to the GO term for the biological process "Proteolys", whereas Ta2291, encoding for an ADP-ribosylation factor (32 in Table 2), was selected because it was homologous to Ta45379, but had a different expression level (18 in Table 2). The selected sequences covered a wide range of expression levels: 11 high (>800 ESTs in each UniGene cluster), 12 moderate (<800/>300 ESTs), 8 low (<300/>100 ESTs) and 1 very low (<100 ESTs) (Table 2).

**Table 2:** List of 32 UniGene clusters and corresponding TCs selected for qRT-PCR analysis of their expression.

**Unigene** **Cluster**	**Gene annotation**	**CV** **TPM**	**NS** **UniGene**	**Rank** **UniGene**	**TIGR contig**	**TIGR** **freq**.	**Rank** **TIGR**
1) *Ta54963*	*GABARAP (GABA-receptor-associated protein)*	9.85	356	1	TC304353, TC304722, TC295997	66/197	29
2) *Ta54171*	*Superoxide dismutase [Cu-Zn]*	11.95	1316	2	TC317053, TC294760, TC297148	110/197	8
3) Ta50503	Ubiquitin	12.79	1088	3	TC278495, TC296829, TC280981	72/197	22
4) *Ta2776*	*Similar to RNase L inhibitor-like protein*	13.17	158	4	TC278756, TC314244	38/197	91
5) *Ta54733*	*Protein of unknown function (DUF 866)*	15.47	167	5	TC335842, TC286574	40/197	86
6) *Ta54948*	*Rab GTPase homolog (Rab7 subfamily)*	16.22	366	6	TC278352, TC303984	33/197	110
7) *Ta30797*	*Similar to phosphogluconate dehydrogenase*	17.27	138	9	TC279294, TC284282	25/197	143
8) *Ta35284*	*Protein transport protein Sec23A*	17.95	101	10	TC278525	31/197	116
9) *Ta54447*	*Acetyl-CoA acyltransferase*	18.47	365	13	TC279279, TC283613	65/197	32
10) *Ta54448*	*Protein of unknown function (DUF52 family)*	19.24	99	15	TC279558	36/197	96
11) Ta 22845	ATP-depend. 26S proteasome regulatory sub.	20.48	186	24	TC353778	64/197	33

12) Ta659	Translation elongation factor 1 alpha-subunit	29.67	3026	94	TC288390	139/197	1
13) Ta30768	GAPDH (Glyceraldehyde-3-phosph. dehydrog.)	22.68	1807	32	TC304401, TC348958, TC310664, TC326825	137/197	2
14) *Ta53919*	*S-adenosylmethionine decarboxylase*	24.73	2533	47	TC320440, TC279487	135/197	3
15) Ta35497	Peptidyl-prolyl cis-trans isomerase (Cyclophilin)	30.81	2131	107	TC348130, TC348051, TC352472	135/197	4
16) Ta53937	Cytosolic malate dehydrogenase	24.58	1368	45	TC300803, TC279938	127/197	5
17) *Ta53891*	*Zn-finger, A20-like domain containing protein*	25.19	1291	50	TC293469	126/197	6
18) *Ta45379*	*ADP-ribosylation factor*	24.90	1180	48	TC337136	118/197	7
19) *Ta1698*	*NADP-isocitrate dehydrogenase*	24.39	761	42	TC283369, TC282416	95/197	10
20) Ta27771	60S ribosomal protein L18a-1	31.94	580	120	TC363506, TC298941	94/197	11
21) Ta53964	Translation elongation factor EF-1alpha (GTPase)	26.88	1074	68	TC279148	89/197	12
22) *Ta54227*	*Cell div. control prot. (AAA-superfam. ATPases)*	24.65	670	46	TC308517, TC281050	87/197	13
23) Ta54280	Translation initiation factor SuI1 family protein	23.53	609	36	TC297312, TC338691	82/197	15
24) *Ta54512*	*Cytochrome b5 family protein*	21.70	695	29	TC284040, TC283036, TC284818	77/197	17
25) *Ta4045*	*Ubiquinol-cytochrome C reduct. iron-sulfur sub*.	23.76	384	37	TC350975, TC327386	77/197	18
26) *Ta53889*	*Hypothetical protein*	29.95	603	97	TC317382, TC333515	74/197	19
27) *Ta53967*	*Vacuolar ATP synthase 16 kDa proteolipid sub*.	25.00	719	49	TC292895, TC344391	71/197	23

28) Ta44405	Beta-tubulin 4 (Tubb4)	22.94	114	33	TC278209	20/197	167
29) Ta54238	Ubiquitin-conjugating enzyme E2	28.67	165	86	TC295800, TC289972, TC289612	57/197	47
30) Ta38797	Similar to histone H3 (Arabidopsis)	33.94	1066	137	TC290356, TC280404, TC313660	68/197	27

31) *Ta55512*	*Cathepsin B-like cysteine protease*	24.55	171	43	TC311052, TC278654	52/197	53
32) *Ta2291*	*ADP-ribosylation factor*	38.31	377	162	TC278558, TC278370	66/197	31

Italics = novel reference genes; normal = traditional reference genes.

CV/TPM = Coefficient of Variation calculated on the expression values (TPMs) in six tissues (flower, inflorescence, leaf, root, seed and stem), UniGene Profile Viewer.

NS UniGene = Number of EST sequences included in each UniGene cluster.

Rank UniGene = Ranking of the UniGene clusters on the basis of the CV of their expression values (TPMs) in six wheat tissues.

TIGR frequency = Number of hosting libraries/total number of cDNA libraries, calculated for each TC or group of TCs linked to the selected UniGene clusters.

Rank TIGR = Ranking of the TCs or group of TCs linked to the selected UniGene clusters on the basis of their frequency.

### Specificity and primer efficiency of the qRT-PCR reactions

The expression stabilities of the 32 selected candidate reference genes and of the five distinct homologous genes for each α-tubulin and actin gene families were assessed by qRT-PCR in a set of 24 tissue samples subdivided into two subsets: the first one included 18 samples representing different tissues and developmental stages of wheat, the second one included six samples and consisted of two temperature treatments (4°C and 33°C), each for 24 and 48 h, and controls. The effect of temperature stresses was checked by RT-PCR analyses of the cold- and heat-responsive genes *wcor14 *and *TaHSP101B *(Additional file 7). Accumulation of *wcor14 *transcripts was undetectable in seedlings grown at 18°C (control samples), whereas strong induction was detected after 24 h of exposition to 4°C, which increased further after 48 h. These results are in perfect agreement with those obtained by Tsetanov et al. [41], who showed by northern analyses of RNA from 23 day old seedlings of *T. aestivum *that transcripts of *wcor14 *accumulated within 3–6 h of cold acclimation at 4°C and reached a maximum after three days. Exposure to 33°C for 24 and 48 h did not induce detectable amount of *wcor14 *transcripts (Additional file 7), confirming its specific induction by low temperatures [41]. In agreement with the results obtained by Campbell et al. [43] and Gulli et al. [44], who analysed the expression of *TaHSP101B *in bread and durum wheats, respectively, our RT-PCR analyses showed that transcripts of these gene were not present in control seedlings grown at 18°C, strong expression was detected after exposition to 33°C for 24 h, then it decreased at very low levels after 48 h of heat treatment (Additional file 7). The absence of *TaHSP101B *transcripts in seedlings exposed to cold temperature (Additional file 7) confirmed that its expression was specifically induced by heat shock.

Total RNA was isolated from the 24 samples, DNase I digested and reverse transcribed.

**Figure 1:**
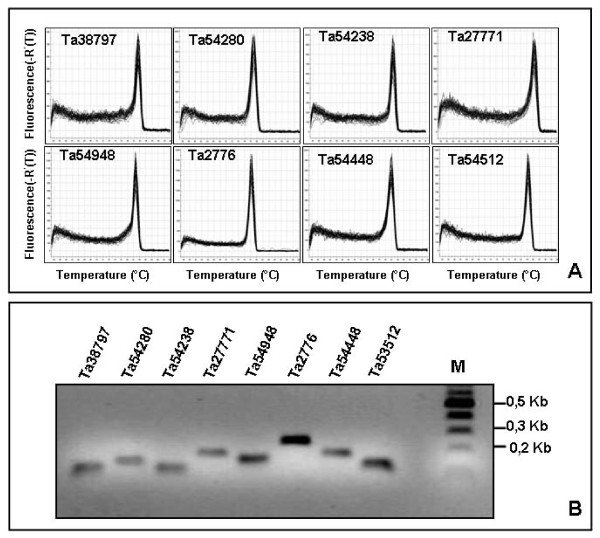
**Specificity of qRT-PCR amplification**. (A) Dissociation curves of six representative reference genes showing single peaks (each including three technical replicates for each of 18 cDNA pools from different tissues and developmental stages of wheat). (B) Agarose gel (2%) showing amplification of a specific PCR product of the expected size for the same genes in (A). Dissociation curves and agarose gels for all the 42 candidate reference genes selected are shown in Additional file 8.

Since we chose a two-step qRT-PCR protocol, reverse transcription and PCR-mediated cDNA amplification were carried out by subsequent steps in separate tubes. The two-step protocol was preferred because it would reduce unwanted dimer formation between primers when SYBR Green is used as a detection dye [51]. The RT reaction was primed by oligo-(dT), rather than using random-sequence primers which anneal preferentially to abundant mRNA species. This choice was critical because, as deduced on the basis of the number of EST sequences comprised in the corresponding UniGene cluster, some of the selected candidate reference genes would be expressed at low level. PCR primer pairs for the 32 selected reference candidate genes and for the five members for each of the α-tubulin and actin gene families were designed and preliminarily tested in qRT-PCR reactions from a pool of all available cDNAs (see Methods). In order to avoid unreliable amplification of splice variants and assure uniform RT efficiencies, typically primer pairs were designed to target an amplicon located within the 3' end region of the transcript of interest. For each biological replicate and tissue sample, the same cDNA pool was used for qRT-PCR amplification of the 42 (32+10) analysed genes using the previously tested gene-specific primers. Moreover, qRT-PCR analysis of every gene was performed in triplicate for each of the 24 cDNA pools, along with no template and RT-minus controls. The 42 primer pairs used to amplify the candidate reference genes generated single amplicons of the expected size from the various cDNA pools, as shown by the presence of single bands in agarose gel electrophoresis and by single-peak melting curves of the PCR products obtained after 40 amplification cycles (Fig. 1 and Additional file 8). A detailed analysis of the dissociation curves confirmed the absence of primer dimers and of other products resulting from non-specific amplification (Additional file 8). A more stringent test of PCR specificity was performed by sequencing the amplification products of each of the 42 candidate reference genes. Always the sequence of the PCR product matched that of the target cDNA, thereby confirming the exquisite PCR specificity of the developed primer pairs.

**Figure 2:**
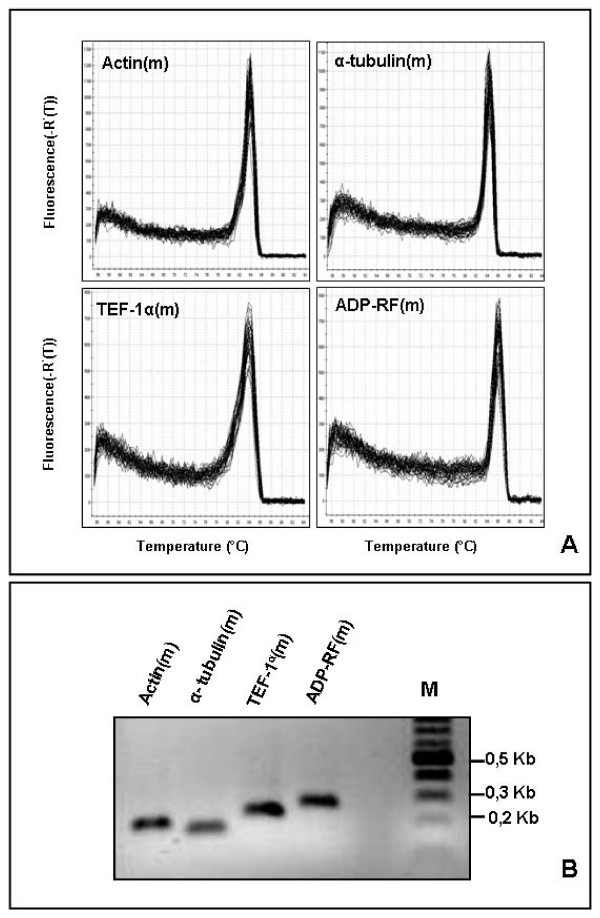
**Dissociation curves (A) and agarose gel of PCR products (B) amplified with the primer pairs designed in conserved regions [Actin (m), α-tubulin (m), TEF-1α (m) and ADP-RF (m)] to amplify transcripts of multiple homologous genes for each of the four families**.

In addition to the primer pairs targeting single transcripts, we set out the evaluation of a further subset of primer pairs, designed in conserved regions identified by sequence comparison, to amplify in a single PCR reaction the transcripts of multiple members of some gene families. Primer pairs designed as TEF-1α (m), ADP-RF (m), Actin (m) and α-tubulin (m), where (m) denotes multiple amplifications, targeted conserved sequences within the transcripts of at least two homologous genes for each family. The simultaneous amplification by conserved primer pairs of sequences transcribed from multiple homologous genes with balanced expression levels has been proposed as a suitable strategy for qRT-PCR normalization. Such an approach has been exploited for the *Arabidopsis *genes *AtACT2 *and *AtACT8*, which display complementary patterns of expression, making their combined expression quasi-constitutive [52]. Gel electrophoresis and melting-curve analysis showed that each of the four conserved primer pairs designed to amplify transcripts from multiple genes of the four analysed families of wheat amplified a PCR product of the expected size from the various cDNA pools (Fig. 2). Single PCR products purified from gels were cloned into the pGEM plasmid vector (Promega) and ten different clones were sequenced for each of the four multiple amplifications. Two sequences of 276 bp and with identity of 90.58% were observed among the ten clones obtained by the PCR with the primer pair ADP-RF (m); also among the ten clones produced by the amplification using the primer pair TEF-1α (m) there were two different sequences of 233 bp with 95.28% identity. In both cases the sequences corresponded to those used for designing the primer pairs within the conserved regions, Ta45379/Ta2291 for ADP-RF and Ta659/Ta53964 for TEF-1α. In each group of ten clones obtained by the primer pairs Actin (m) and α-tubulin (m) three similar homologous sequences were found, which matched three of the five members of their respective gene families (Ta54825/Ta53908/Ta20863 for Actin and Ta53981/Ta33558/Ta54519 for α-tubulin), showing that multiple amplifications were produced also by these conserved primers.

Although fluorescence intensity at a given stage during qRT-PCR is primarily determined by the initial concentration of the cDNA template, its increase is strongly affected by the amplification efficiency. When the efficiency is 100%, the amount of a cDNA amplicon targeted by a given primer pair is doubled at every PCR cycle during the exponential phase; however, several factors can significantly affect the efficiency of individual PCR reactions. In our study the PCR efficiency of each primer pair in each of the two biological replicates was determined by five-point standard curves of a 5-fold dilution series (1:1–1:625) from pooled cDNA. By plotting the C_t_s against the logarithm of the relative amounts of template (serial dilutions from pooled cDNA) a standard curve was generated by the Mx3000PTM software system and the efficiency was computed by the equation E = (10^[-1/m]^-1) × 100, where m is the slope of linear regression model fitted over log-transformed data of the input cDNA concentration versus C_t _values according to the linear equation y = m*log(x) + b. Moreover, the Mx3000PTM software system computed the R^2 ^value (Coefficient of determination), which represents an indicator of the quality of the fit of the standard curve to the plotted data points. The R^2 ^values calculated for all the standard curves in each of the two biological replicates were higher than 0.994, indicating a strong linear relationship between the detected C_t _values and the correspondent relative amount of template in all the amplification reactions (Additional file 9). The efficiencies of all the 46 primer pairs were comprised between 94% and 110%, about 44% (29/46) of them with values close to 100% (97–103%) (Additional file 9), reflecting the high quality of the amplification reactions. Generally the standard deviation of primer efficiencies was low (<1.4), indicating comparable amplification efficiencies between the two biological replicates, only 14 primer pairs showed values higher than the 2% (SD > 1.4). The C_t_s of the three replicates and the efficiency values (Additional file 9) were used to compute the relative expression level of the selected candidate reference genes for each of the two biological replicates.

### Selection of the best candidate reference gene within the same family

In order to select the most stable gene encoding actin, α-tubulin, ADP-ribosylation factor and translation elongation factor, the expression stability of genes belonging to each family was analyzed by single factor ANOVA on the C_t _values of five different data-sets: 1) all 24 samples including tissues, developmental stages and temperature treatments; 2) 18 samples relative to different tissues and developmental stages; 3) six samples consisting of two temperature treatments (4°C and 33°C), each for 24 and 48 h and their controls; 4) six samples relative to the single floral organs from fully emerged spikes; 5) six samples referring to vegetative tissues and developmental stages (shoots, stems and leaves). These data sets were used to evaluate the expression stability of the genes belonging to the four families by 18 primer pairs, 14 of them targeting specific gene transcripts of the gene families (five each for actin and α-tubulin and two each for ADP-ribosylation factor and translation elongation factor) and four primer pairs which amplified multiple transcripts for each of the four gene families [Actin (m), α-tubulin (m), ADP-RF (m) and TEF-1α (m)]. Table 3 reports the CV values and the C_t _differences (ΔC_t _= C_t_max-C_t_min) obtained by the 18 primer pairs for each of the five data-sets considered, whereas the complete statistics obtained by ANOVA analysis are reported in Additional file 10. The CV of the C_t _values gives a straightforward indication of the expression stability of a particular gene; most genes with the lowest CV values showed also the lowest values of C_t _range (ΔC_t_) and MST (Mean Square of Treatments) (Additional file 10). On the other hand, the very low values of the MSE (Mean Square of Error) for all the analysed genes, showed that the C_t _values obtained in different replicates were highly reliable and the qRT-PCR experiments generate highly reproducible results (Additional file 10).

**Table 3:** Coefficient of Variation^a ^(CV) and C_t _difference (ΔC_t _= C_t_max-C_t_min) determined on raw C_t _values obtained by 18 primer pairs which amplified specific or multiple (m) gene transcripts of four gene families (Actin, α-tubulin, Translation elongation factor and ADP-ribosylation factor) in five data-sets.

**Unigene cluster**	**Gene annotation**	**I Data set^b^**		**II Data set^c^**		**III Data set^d^**		**IV Data set^e^**		**V Data set^f^**	
		**CV**	**ΔC_t_**	**CV**	**ΔC_t_**	**CV**	**ΔC_t_**	**CV**	**ΔC_t_**	**CV**	**ΔC_t_**
Ta54225	Actin	3.87	3.01	4.14	2.90	1.83	1.21	1.99	1.14	2.95	1.57
Actin (m)	Actin	4.41	3.78	4.46	3.78	3.87	1.90	1.27	0.65	3.12	1.76
Ta1868	Actin	5.37	4.81	5.87	4.81	2.10	1.26	3.28	2.36	2.67	1.96
Ta4344	Actin	6.09	6.15	6.90	6.15	1.73	1.12	4.35	2.58	6.71	4.97
Ta20863	Actin	6.65	6.50	7.26	6.65	3.03	2.40	3.81	2.43	7.56	6.42
Ta53908	Actin	7.20	6.90	7.92	6.90	2.67	1.94	3.70	2.23	8.20	6.21

Ta25534	α-tubulin 5	4.65	4.14	4.79	4.14	3.16	2.25	2.75	1.90	2.98	2.31
Ta54519	α-tubulin 4	6.94	7.33	7.83	7.33	4.03	2.82	5.65	3.70	7.04	5.38
α-tubulin (m)	α-tubulin	7.01	6.42	7.82	6.42	3.31	1.63	3.84	1.61	8.25	5.41
Ta53981	α-tubulin 2	7.54	6.39	8.31	6.39	4.00	2.04	3.52	1.93	9.21	6.25
Ta33558	α-tubulin 3	7.77	7.02	8.59	7.02	4.09	2.56	5.21	3.52	9.37	7.02
Ta5566	α-tubulin 1	8.05	8.49	8.82	8.49	4.84	2.73	5.42	3.58	8.04	6.60

Ta53964	Translation EF 1α-subunit	3.55	3.46	3.74	3.46	1.04	0.66	1.74	0.91	2.71	2.00
TEF-1α (m)	Translation EF 1α-subunit	4.53	4.09	5.08	4.09	2.20	1.17	2.77	1.49	4.01	2.60
Ta659	Translation EF 1α-subunit	4.82	4.35	5.27	4.35	2.12	1.16	2.50	1.22	3.82	2.48

Ta2291	ADP-ribosylation factor	4.54	3.46	4.85	3.46	2.02	1.27	1.38	0.70	3.86	2.55
Ta45379	ADP-ribosylation factor	4.59	4.03	4.85	4.03	2.21	1.35	2.15	1.18	5.16	3.77
ADP-RF (m)	ADP-ribosylation factor	6.70	4.68	6.24	4.68	3.73	2.23	2.22	1.19	6.26	4.34

^a^CV = Standard deviation divided by mean and multiplied by 100 of MST (Mean Square Total) among tissues, biological experiments and technical replicates within each of the two experiments.

^b^All the 24 samples including tissues, developmental stages and temperature treatments.

^c^18 tissues and developmental stages.

^d^Six samples consisting of two temperature treatments (4°C and 33°C) for 24 and 48 h and their controls.

^e^Six floral organs from fully emerged spikes.

^f^Six vegetative tissues and developmental stages (shoots, stems and leaves).

Table 3 and Additional file 10 show that the most stable genes (lowest CV values) for each family were: the gene corresponding to the UniGene cluster Ta25534 for α-tubulin, Ta54825 for actin, Ta2291 for ADP-ribosylation factor and Ta53964 for translation elongation factor. These four genes were chosen as representative of the corresponding families and used for further comparison with the other candidate reference genes.

### Expression levels of the selected candidate reference genes

**Figure 3:**
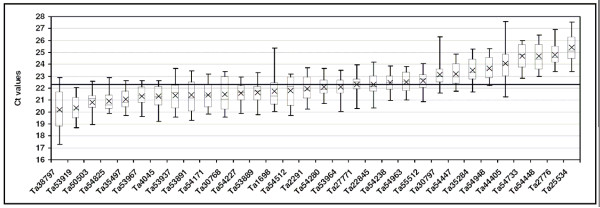
**Expression level of 32 candidate reference genes in 24 tissues, developmental stages and seedlings exposed to high and low temperatures**. The expression levels (C_t _values) of each candidate reference gene (UniGene cluster) are shown as average (diagonal cross), median (horizontal lines), 25^th ^to 75^th ^percentile (boxes) and expression ranges (whiskers) for the 24 cDNA pools analysed. The line at C_t _22.31, corresponding to the general mean, discriminates the reference genes with high and low expression.

In order to avoid possible errors due to the interaction of co-regulated genes, further analyses were carried out on 32 genes involved in distinct biological processes and metabolic pathways; they comprised 28 genes selected on the basis of their uniform expression detected in UniGene and TIGR and a single gene for each of the four gene families. The expression levels could be grouped into two arbitrary categories: 19 highly expressed genes, whose mean C_t _values were below the general mean (C_t _= 22.31 cycles) and 13 lowly expressed genes, with mean C_t _values above this value (Fig. 3). The highest expression was shown by Ta38797 (Histone), Ta53919 (S-adenosylmethionine decarboxylase), Ta50503 (Ubiquitin) and Ta54825 (Actin), with mean C_t _values between 20 and 21 cycles, the lowest by Ta25534 (α-tubulin), Ta2776 (RNase L inhibitor-like protein), Ta54448 and Ta54733 (Proteins of unknown function), and Ta44405 (β-tubulin), with mean C_t _values between 26 and 24 cycles (Fig. 3). On the basis of the number of EST sequences included in each Unigene cluster, the 19 reference genes with low C_t _values had been previously classified either as highly expressed (9 genes with more than 800 EST) and moderately expressed (10 genes with more than 300 EST). Of the 13 reference genes with low C_t _values, only three including more than 300 ESTs (Ta54963, Ta54447 and 54948) were considered moderately expressed in UniGene, whereas the expression level of the remaining 10 genes was low (<300/>100 ESTs) or very low (<100 ESTs). In our opinion, these data indicate a substantial agreement between the gene expression levels evaluated by qRT-PCR analyses and those estimated by the number of ESTs included in each corresponding UniGene cluster. Ta54963 (GABA-receptor associated protein), Ta54238 (Ubiquitin-conjugating enzyme), Ta35497 (Cyclophilin), Ta53967 (Vacuolar ATP synthase 16 kDa proteolipid subunit) exhibited the lowest gene expression variation (less than 3 cycles), while Ta44405 (β-tubulin), Ta38797 (Histone) and Ta1698 (NADP-isocitrate dehydrogenase) showed the most variable expression (more than 5 cycles).

### Expression stability of the selected candidate reference genes

As discussed in the previous paragraph, it was evident that none of the 32 selected candidate reference genes showed a constant expression under all the tested conditions (tissues, developmental stages and temperature stress) in wheat. Hence, as expected, it was not possible to find an all purpose reference gene, and it would be necessary to select the most suitable one for normalizing gene expression considering the peculiar and specific experimental conditions. The relationships between expression stability of the 32 candidate genes and different types of tissues and/or experimental conditions were tested by running the programs geNorm [13] and NormFinder [15] using the five data-sets previously described.

**Figure 4:**
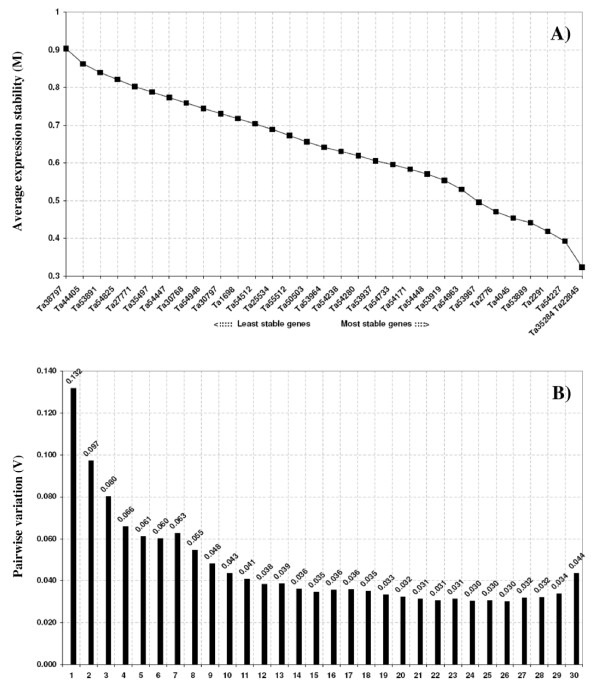
**geNorm output charts of M and V values for the 32 selected reference genes in 24 samples**. (A) Stepwise exclusion of the least stable genes (lowest average expression stability measure M). The x-axis from left to right indicates the ranking of the genes according to their expression stability (M). (B) Determination of the optimal number of control genes for accurate normalization calculated on the basis of pair-wise variation (V) analysis. 1 = V2/3...........30 = V31/32.

The program geNorm computes the expression stability M of a gene based on the average pairwise variation between all studied genes. The lowest M values are produced by genes with the most stable expression. Successive elimination of the least stable genes generates their ranking according to the M values and results in the identification of the two most stable reference genes. The average expression M values of the 32 candidate reference genes for all the 24 samples (I Data set) tested in our study are plotted in figure 4A, whereas those for the other four data-sets (II-V) are shown in the figures reported in the additional file 11. All the 32 genes showed high expression stability and had M values lower than 0.9, below the default limit of 1.5 suggested by the geNorm program. The curves represent the stepwise exclusion of the least stable candidate reference gene. When the complete data set including 24 samples was analysed (Fig. 4A), Ta38797 (Histone) and Ta44405 (β-tubulin) were the first excluded as the least stable genes (highest M values of 0.90 and 0.86). Ta35284 (Protein transport protein Sec23A) and Ta22845 (ATP-dependent 26S proteasome regulatory subunit), both with M = 0.32, were identified as the most stable pair of genes. The ranking order of the following eight most stable genes, with M values comprised between 0.39 and 0.55, was: Ta54227 (3^rd^, Cell division control protein, AAA-superfamily of ATPases), Ta2291 (4^th^, ADP-ribosylation factor), Ta53899 (5^th^, Hypothetical protein), Ta4045 (6^th^, Ubiquinol-cytochrome C reductase iron-sulfur subunit), Ta2776 (7^th^, RNase L inhibitor-like protein), Ta53967 (8^th^, Vacuolar ATP synthase 16 kDa subunit), Ta54963 (9^th^, GABA-receptor-associated protein) and Ta53919 (10^th^, S-adenosylmethionine decarboxylase) (Fig. 4A). The ranking remained very similar when the set of 18 samples, including different tissues and developmental stages, was analysed, with the lowest M value (0.33) for Ta35284 and Ta22845 (Figure A in additional file 11). The rank order of the following seven most stable genes was the same obtained analysing the whole data set of 24 samples (compare figures 4A and A of additional file 11), and the least stable gene with the highest M value (0.87) was Ta44405 (β-tubulin). In the temperature treatment series Ta54825 (Actin) and Ta53889 (Hypothetical protein), both with M = 0.071, were the most stable genes, followed by Ta22845 (3^rd^, ATP-dependent 26S proteasome regulatory subunit), Ta2291 (4^th^, ADP-ribosylation factor), Ta54238 (5^th^, Ubiquitin-conjugating enzyme), Ta54948 (6^th^, Rab GTPase homolog, Rab7 subfamily), Ta2776 (7^th^, RNase L inhibitor-like protein), Ta30797 (8^th^, Phosphogluconate dehydrogenase), Ta54227 (9^th^, Cell division control protein, AAA-superfamily of ATPases) and Ta4045 (10^th^, Ubiquinol-cytochrome C reductase iron-sulfur subunit) (Fig. B in additional file 11). Seven of the top ten most stable candidate reference genes in the data set of 18 samples, (Ta53889, Ta22845, Ta2291, Ta54948, Ta2776, Ta54227 and Ta4045) were also classified among the ten most stable genes in the temperature treatment series, where the two least stable genes were Ta53891 (Zn-finger, A20-like domain containing protein) and Ta44405 (β-tubulin) (Fig. B in additional file 11). In the six samples relative to the single floral organs from fully emerged spikes Ta2776 (RNase L inhibitor-like protein) and Ta35284 (Protein transport protein Sec23A) were the most stable genes (M value 0.18) and Ta54512 (Cytochrome b5 family protein) the least stable (M value 0.72) (Fig. C in additional file 11). Finally, in the six samples comprising only vegetative tissues Ta4045 (Ubiquinol-cytochrome C reductase iron-sulfur subunit) and Ta50503 (Ubiquitin) were the most stable (lowest M value 0.09), followed by Ta53899 (3^rd^, Hypothetical protein), Ta54227 (4^th^, Cell division control protein, AAA-superfamily of ATPases), Ta54238 (5^th^, Ubiquitin-conjugating enzyme E2), Ta53891 (6^th^, Zn-finger, A20-like domain containing protein), Ta54447 (7^th^, Acetyl-CoA acyltransferase), Ta2291 (8^th^, ADP-ribosylation factor), Ta53919 (9^th^, S-adenosylmethionine decarboxylase) and Ta55512 (10^th^, Cathepsin B-like cysteine protease), whereas Ta1698 (NADP-isocitrate dehydrogenase) and Ta44405 (β-tubulin), with M values of 0.63 and 0.59, were the least stable genes (Fig. D in additional file 11).

The high expression stability of a gene indicates that the use of a single internal control is appropriate, but some studies require the normalization based on two or more stable internal control genes. The geNorm program, in addition to the gene stability measure M, computes a normalization factor (NF) and assesses the optimal number of reference genes for generating that factor. The pairwise variations Vn/Vn+1 between two sequential normalization factors (NFn and NFn+1) are used to determine the necessity of adding the next more stable control gene for reliable normalization. Pairwise variations were calculated using geNorm for each data set to determine the optimal number of internal control genes for normalization (Fig. 4B and Additional file 12). In all the five data sets analysed the two most stable candidate reference genes yielded V values lower than 1.5. That cut-off was suggested as the limit beneath which it would not be necessary to include additional reference genes for normalization [13]. Since the V values did not significantly decrease when more than four reference genes were included (Fig. 4B and Additional file 12), it can be concluded that normalization using either the two or three most stable reference genes would be an adequate normalization criterion for gene profiling studies in the five data sets considered.

NormFinder (the second adopted program based on a model approach) ranks the best candidate references genes according to their minimal combined inter- and intra-group variation of expression. The results obtained by NormFinder analysis for the five data sets are shown in Table 4. When the complete data set of 24 samples was considered, Ta54227 (Cell division control protein, AAA-superfamily of ATPases) was identified as the most stable gene, with a stability value of 0.035, followed by Ta2291 (2^nd^, ADP-ribosylation factor), Ta2776 (3^rd^, RNase L inhibitor-like protein), Ta35284 (4^th^, Protein transport protein Sec23A), Ta53919 (5^th^, S-adenosylmethionine decarboxylase), Ta53967 (6^th^, Vacuolar ATP synthase 16 kDa proteolipid subunit), Ta54171 (7^th^, Superoxide dismutase), Ta54963 (8^th^, GABA-receptor-associated protein), Ta54733 (9^th^, Protein of unknown function, DUF 866) and Ta4045 (10^th^, Ubiquinol-cytochrome C reductase iron-sulfur subunit) (stability values between 0.049 and 0.069); the two least stable genes were Ta44405 (β-tubulin) and Ta38797 (Histone), with stability values of 0.150 and 0.198 respectively, the same identified as the worst genes by geNorm. The best combination of two genes suggested by NormFinder was that of Ta54227 and Ta2291, which improved the stability value to 0.030, indicating a more reliable normalization than that based on single genes. Similar results were obtained for 18 samples including tissues/organs at different developmental stages of wheat, with the lowest stability value (0.032) for Ta54227 (Table 4). In the temperature treatment series NormFinder selected Ta30797 (Phosphogluconate dehydrogenase), with a stability value of 0.030, as the most stable single gene, whereas, as in geNorm analysis, Ta44405 (β-tubulin) and Ta53891 (Zn-finger, A20-like domain containing protein) were identified as the two least stable genes (Table 4). The best combination of two genes was that of Ta30797 and Ta4045, with a stability value of 0.023 (Table 4). In the six samples relative to the single floral organs NormFinder identified as the best combination of two genes that of Ta30797 and Ta54227, with stability value of 0.045 which was significantly lower than that (0.061) of the most stable gene (Ta30797) taken alone. Finally, in the six samples including only vegetative tissues Ta54227 showed again the lowest stability value (0.022), the same value calculated for the best combination of two genes (Ta54227 and Ta53889).

**Table 4:** Candidate reference genes and best combination of two genes (BCTG) listed according to their expression stability calculated by NormFinder in five data sets.

	**I Data set**			**II Data set**			**III Data set**			**IV Data set**			**V Data set**	
									
	**UniGene Cluster**	**Stabil. value**		**UniGene Cluster**	**Stabil. value**		**UniGene Cluster**	**Stabil. value**		**UniGene Cluster**	**Stabil. value**		**UniGene Cluster**	**Stabil. value**
1)	Ta54227	0.035	1)	Ta54227	0.032	1)	Ta30797	0.030	1)	Ta30797	0.061	1)	Ta54227	0.022
2)	Ta2291	0.049	2)	Ta2291	0.064	2)	Ta4045	0.033	2)	Ta54227	0.065	2)	Ta53889	0.038
3)	Ta2776	0.055	3)	Ta54948	0.064	3)	Ta54948	0.038	3)	Ta1698	0.069	3)	Ta50503	0.051
4)	Ta35284	0.057	4)	Ta2776	0.072	4)	Ta54238	0.046	4)	Ta2776	0.072	4)	Ta4045	0.054
5)	Ta53919	0.063	5)	Ta54448	0.073	5)	Ta2291	0.048	5)	Ta53967	0.081	5)	Ta54238	0.072
6)	Ta53967	0.064	6)	Ta35284	0.074	6)	Ta1698	0.052	6)	Ta54963	0.082	6)	Ta55512	0.088
7)	Ta54171	0.066	7)	Ta53919	0.077	7)	Ta54733	0.054	7)	Ta54948	0.082	7)	Ta53967	0.091
8)	Ta54963	0.067	8)	Ta54171	0.080	8)	Ta22845	0.054	8)	Ta54448	0.084	8)	Ta54448	0.091
9)	Ta54733	0.068	9)	Ta53967	0.080	9)	Ta2776	0.063	9)	Ta22845	0.098	9)	Ta53919	0.095
10)	Ta4045	0.069	10)	Ta35497	0.081	10)	Ta35284	0.068	10)	Ta44405	0.099	10)	Ta53937	0.096
11)	Ta54448	0.069	11)	Ta54733	0.082	11)	Ta53937	0.073	11)	Ta53891	0.102	11)	Ta35284	0.098
12)	Ta22845	0.073	12)	Ta54963	0.084	12)	Ta54963	0.074	12)	Ta54238	0.103	12)	Ta2776	0.101
13)	Ta53937	0.074	13)	Ta4045	0.090	13)	Ta54512	0.080	13)	Ta54280	0.106	13)	Ta2291	0.102
14)	Ta53889	0.075	14)	Ta53899	0.093	14)	Ta54171	0.084	14)	Ta35284	0.108	14)	Ta54447	0.106
15)	Ta54238	0.077	15)	Ta54280	0.093	15)	Ta53967	0.086	15)	Ta54171	0.114	15)	Ta53891	0.107
16)	Ta54280	0.080	16)	Ta22845	0.096	16)	Ta54825	0.088	16)	Ta53937	0.124	16)	Ta53964	0.107
17)	Ta50503	0.084	17)	Ta54238	0.097	17)	Ta53889	0.093	17)	Ta25534	0.137	17)	Ta53948	0.107
18)	Ta53964	0.089	18)	Ta53937	0.097	18)	Ta54447	0.099	18)	Ta53919	0.145	18)	Ta35497	0.109
19)	Ta54512	0.092	19)	Ta53964	0.103	19)	Ta53919	0.103	19)	Ta2291	0.148	19)	Ta22845	0.113
20)	Ta55512	0.095	20)	Ta50503	0.106	20)	Ta54227	0.105	20)	Ta54733	0.155	20)	Ta38797	0.114
21)	Ta25534	0.100	21)	Ta54512	0.113	21)	Ta50503	0.123	21)	Ta35497	0.158	21)	Ta30768	0.119
22)	Ta1698	0.101	22)	Ta25534	0.117	22)	Ta55512	0.144	22)	Ta50503	0.167	22)	Ta25534	0.121
23)	Ta30797	0.104	23)	Ta55512	0.119	23)	Ta30768	0.168	23)	Ta53964	0.181	23)	Ta54512	0.136
24)	Ta54948	0.105	24)	Ta30797	0.129	24)	Ta54280	0.173	24)	Ta38797	0.182	24)	Ta54733	0.152
25)	Ta30768	0.110	25)	Ta30768	0.132	25)	Ta35497	0.177	25)	Ta30768	0.187	25)	Ta27771	0.157
26)	Ta54447	0.113	26)	Ta1698	0.134	26)	Ta54448	0.181	26)	Ta54825	0.219	26)	Ta30797	0.170
27)	Ta35497	0.116	27)	Ta38797	0.138	27)	Ta53964	0.184	27)	Ta55512	0.223	27)	Ta54171	0.172
28)	Ta27771	0.118	28)	Ta53891	0.141	28)	Ta27771	0.195	28)	Ta53889	0.247	28)	Ta54963	0.173
29)	Ta54825	0.135	29)	Ta54447	0.142	29)	Ta25534	0.201	29)	Ta27771	0.250	29)	Ta54280	0.197
30)	Ta53891	0.135	30)	Ta54825	0.144	30)	Ta38797	0.207	30)	Ta54447	0.254	30)	Ta54825	0.208
31)	Ta44405	0.150	31)	Ta27771	0.146	31)	Ta44405	0.304	31)	Ta4045	0.255	31)	Ta44405	0.303
32)	Ta38797	0.198	32)	Ta44405	0.171	32)	Ta53891	0.352	32)	Ta54512	0.295	32)	Ta1698	0.322
														
	BCTG			BCTG			BCTG			BCTG			BCTG	
	Ta54227+Ta2291	0.030		Ta54227+Ta2291	0.032		Ta30797+Ta4045	0.023		Ta30797+Ta54227	0.045		Ta54227+Ta53889	0.022

The stability ranking of the reference genes across the five analysed data sets obtained by geNorm and NormFinder was not identical for the genes with the most stable expression (Table 5). In particular, the best normalization genes selected by the two programs were not the same, but there was substantial agreement when the groups of genes with the most and least stable expression were considered. For instance, in the whole data set of 24 samples (I Data set in Table 5), the most stable genes identified by NormFinder (Ta54227 and Ta2291) were classified as the third and fourth most stable genes by geNorm, and out of the top ten most stable reference genes determined by NormFinder, eight were also classified among the ten most stable genes by geNorm (Table 5). Moreover, the ranking of the eleven least stable genes determined by NormFinder was the same of geNorm (Table 5). Similar relationships among the two methods were also observed by comparing the ranking of the 32 candidate reference genes in the remaining four data sets (Table 5).

**Table 5:** Ranking of candidate reference genes according to their expression stability assessed by GeNorm and NormFinder in five data sets.

**Rank**	**GeNorm**					**Rank**	**NormFinder**				
			
	**I Data set**	**II Data set**	**III Data set**	**IV Data set**	**V Data set**		**I Data set**	**II Data set**	**III Data set**	**IV Data set**	**V Data set**
			
1/2	Ta35284/Ta22845	Ta35284/Ta22845	Ta54825/Ta53889	Ta2776/35284	Ta4045/Ta50503	1/2	Ta54227/Ta2291	Ta54227/2291	Ta30797/Ta4045	Ta30797/Ta54227	Ta54227/Ta53889

3	Ta54227	Ta54227	Ta22845	Ta54227	Ta53889	3	Ta2776	Ta54948	Ta54948	Ta1698	Ta50503
4	Ta2291	Ta2291	Ta2291	Ta1698	Ta54227	4	Ta35284	Ta2776	Ta54238	Ta2776	Ta4045
5	Ta53889	Ta53889	Ta54238	Ta53967	Ta54238	5	Ta53919	Ta54448	Ta2291	Ta53967	Ta54238
6	Ta4045	Ta4045	Ta54948	Ta22845	Ta53891	6	Ta53967	Ta25284	Ta1698	Ta54963	Ta55512
7	Ta2776	Ta2776	Ta2776	Ta54448	Ta54447	7	Ta54171	Ta53919	Ta54733	Ta54948	Ta53967
8	Ta53967	Ta54448	Ta30797	Ta2291	Ta2291	8	Ta54963	Ta54171	Ta22845	Ta54448	Ta54448
9	Ta54963	Ta54948	Ta54227	Ta54280	Ta53919	9	Ta54733	Ta53967	Ta2776	Ta22845	Ta53919
10	Ta53919	Ta53967	Ta4045	Ta54963	Ta55512	10	Ta4045	Ta35497	Ta35284	Ta44405	Ta53937
11	Ta54448	Ta54171	Ta35284	Ta30797	Ta53967	11	Ta54448	Ta54733	Ta53937	Ta53891	Ta35284
12	Ta54171	Ta53919	Ta54733	Ta54948	Ta54448	12	Ta22845	Ta54963	Ta54963	Ta54238	Ta2776
13	Ta54733	Ta54963	Ta1698	Ta54238	Ta53937	13	Ta53937	Ta4045	Ta54512	Ta54280	Ta2291
14	Ta54937	Ta35497	Ta53937	Ta53891	Ta35284	14	Ta53889	Ta53889	Ta54171	Ta35284	Ta54447
15	Ta54280	Ta54733	Ta50503	Ta44405	Ta54948	15	Ta54238	Ta54280	Ta53967	Ta54171	Ta53891
16	Ta54238	Ta53964	Ta54963	Ta25534	Ta2776	16	Ta54280	Ta22845	Ta54825	Ta53937	Ta53964
17	Ta53964	Ta54280	Ta54512	Ta53937	Ta22845	17	Ta50503	Ta54238	Ta53889	Ta25534	Ta54948
18	Ta50503	Ta53937	Ta53967	Ta54171	Ta53964	18	Ta53964	Ta53937	Ta54447	Ta53919	Ta35497
19	Ta55512	Ta54238	Ta54171	Ta53919	Ta35497	19	Ta54512	Ta53964	Ta53919	Ta2291	Ta22845
20	Ta25534	Ta50503	Ta53919	Ta54733	Ta38797	20	Ta55512	Ta50503	Ta54227	Ta54733	Ta38797
21	Ta54512	Ta25534	Ta54447	Ta35497	Ta25534	21	Ta25534	Ta54512	Ta50503	Ta35497	Ta30768
22	Ta1698	Ta54512	Ta55512	Ta53964	Ta30768	22	Ta1698	Ta25534	Ta55512	Ta50503	Ta25534
23	Ta30797	Ta55512	Ta54280	Ta50503	Ta54512	23	Ta30797	Ta55512	Ta30768	Ta53964	Ta54512
24	Ta54948	Ta30797	Ta35497	Ta38797	Ta54733	24	Ta54948	Ta30797	Ta54280	Ta38797	Ta54733
25	Ta30768	Ta30768	Ta27771	Ta30768	Ta27771	25	Ta30768	Ta30768	Ta35497	Ta30768	Ta27771
26	Ta54447	Ta53891	Ta53964	Ta54825	Ta54963	26	Ta54447	Ta1698	Ta54448	Ta54825	Ta30797
27	Ta35497	Ta54447	Ta38797	Ta55512	Ta30797	27	Ta35497	Ta38797	Ta53964	Ta55512	Ta54171
28	Ta27771	Ta1698	Ta30768	Ta53889	Ta54171	28	Ta27771	Ta53891	Ta27771	Ta53889	Ta54963
29	Ta54825	Ta38797	Ta54448	Ta4045	Ta54280	29	Ta54825	Ta54447	Ta25534	Ta27771	Ta54280
30	Ta53891	Ta27771	Ta25534	Ta27771	Ta54825	30	Ta53891	Ta54825	Ta38797	Ta54447	Ta54825
31	Ta44405	Ta54825	Ta44405	Ta54447	Ta44405	31	Ta44405	Ta27771	Ta44405	Ta4045	Ta44405
32	Ta38797	Ta44405	Ta53891	Ta54512	Ta1698	32	Ta38797	Ta44405	Ta53891	Ta54512	Ta1698

### The choice of the reference genes affects the normalization of a gene of interest

The expression levels of the target gene TaPDIL1-1 were used as an example to show the effect of using different normalization genes on estimating its relative expression. TaPDIL1-1 encodes the typical PDI (Protein Disulfide Isomerase) which accomplishes several metabolic functions, the most important consisting in disulfide bond formation and isomerization during the folding of secretory proteins [39,40]. Previous Northern and RT-PCR analyses had shown the constitutive expression of PDI in several wheat tissues, but a much higher expression during the early stages of wheat seed development [39,40]. The transcript quantity of TaPDIL1-1 was determined by qRT-PCR in the same 24 cDNA samples used for selection of reference genes. Moreover the relative expression level of the gene of interest was calculated in the samples of four data sets: 1) 18 different tissues and developmental stages of wheat; 2) six vegetative tissues and developmental stages (shoots, stems and leaves); 3) six floral organs from fully emerged spikes; 4) six samples consisting of two temperature treatments (4°C and 33°C), each for 24 and 48 h, and their controls. The TaPDIL1-1 expression was normalized in each of the four data sets following five different methods: i) geometric average of the three genes selected by geNorm; ii) geometric average of the two genes selected by NormFinder; iii) top-ranking genes identified by NormFinder; iv) gene related to the Unigene cluster Ta54825 (Actin); v) gene related to the Unigene cluster Ta25534 (α-tubulin). Since members the actin and α-tubulin gene families have been commonly used as controls for the normalization of gene expression in wheat, Ta54825 and Ta25534 were used as examples of the consequences of using unstable reference genes.

**Figure 5:**
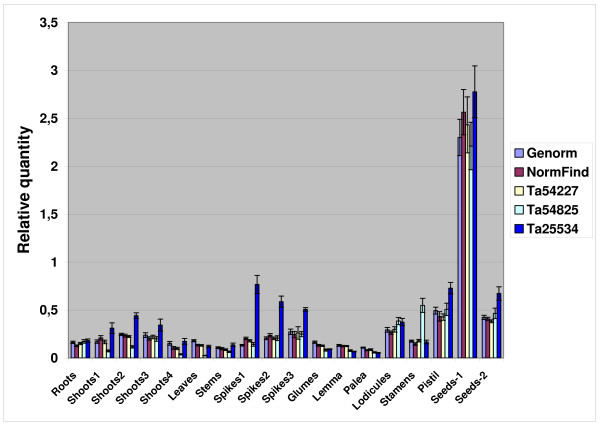
**Relative quantification of TaPDIL1-1 expression using selected reference genes as internal controls in 18 tissues and developmental stages of wheat**. 1) Roots from plants with single shoot and three leaves unfolded (Feekes scale 1.3); 2) Shoots1 = single shoots and leaves from plants at Feekes scale 1.3; 3) Shoots2 = shoots at the beginning of tillering (Feekes scale 2); 4) Shoots3 = shoots from plants with formed tillers (Feekes scale 3); 5) Shoots4 = shoots at the beginning of erect growth (Feekes scale 4); 6) flag leaves at booting stage (Feekes scale 10); 7) stems at booting stage (Feekes scale 10); 8–10) Spikes1-3 = spikes collected at intervals of 10–12 days (three developmental stages: 15–20 mm, flag leaf unfolding and heading stage); 11–16) single floral organs (glumes, lemma, palea, lodicules, stamens and pistil) from fully emerged spikes (Feekes scale 10.5); 17–18) Seeds 1–2 = seeds collected 15 (medium milk stage) and 30 (hard dough stage) days after anthesis. Normalization was performed using different methods (geNorm: geometric average of the three most stable reference genes; NormFind: geometric average of the two most stable genes; Ta54227: the most stable gene identified by NormFinder; Ta54825 = Actin; Ta25534 = α-tubulin). Normalized values of TaPDIL1-1 relative expression are given as average ± SD.

**Figure 6:**
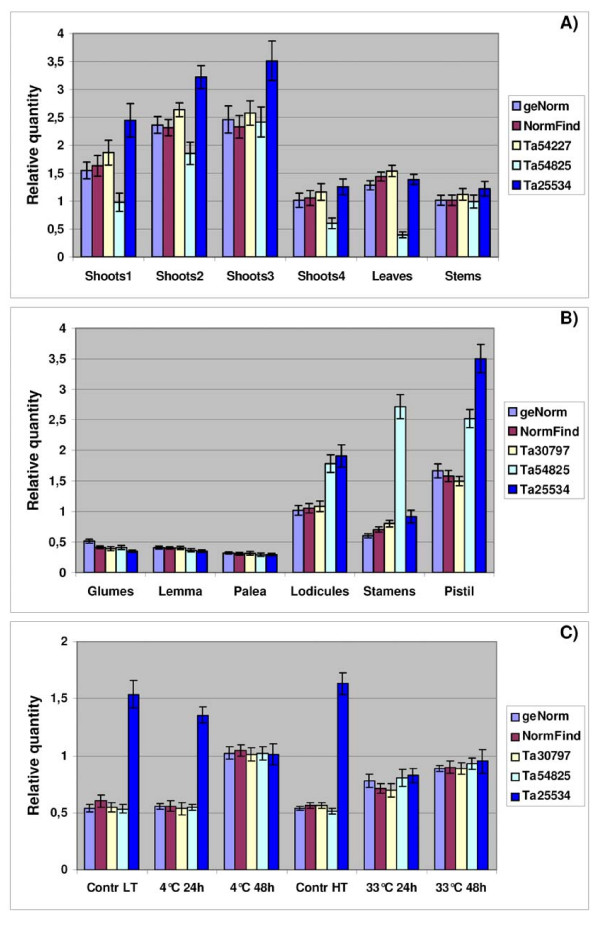
**Relative quantification of TaPDIL1-1 transcripts in three data sets using selected reference genes as internal controls**. A) Six vegetative tissues and shoot developmental stages (shoots, stems and leaves); B) six floral organs from fully emerged spikes; C) six samples consisting of two temperature treatments (4°C and 33°C) for 24 and 48 h and their controls (LT = low temperature; HT = high temperature). The TaPDIL1-1 expression levels were normalized using the five methods described in Fig. 5. Normalized values of TaPDIL1-1 relative expression are given as average ± SD.

Normalization of the expression of TaPDIL-1-1 using the reference genes selected by the three methods based on geNorm and NormFinder programmes did not detect significant differences in the relative expression of the gene of interest in all the four analysed data sets (Figs. 5 and 6). As expected, the mRNA of TaPDIL1-1 was constitutively expressed in all analysed tissues, with very high transcription at the early stage of seed development (Seeds-1) and moderately high expression also in the middle and late stages of spike development (Spikes 2–3) and in floral organs such as lodicules and pistil. The lowest transcription was detected in vegetative tissues, such as mature leaves and stems, and in the non-reproductive floral organs (glumes, lemma and palea) (Fig. 5). On the contrary, normalization of the TaPDIL1-1 transcription levels by the commonly used reference genes Ta54825 (Actin) and Ta25534 (α-tubulin) led to significant over- or under-estimation of transcription of the gene of interest (Fig. 5). Normalization using Ta25534 (α-tubulin) of the TaPDIL-1 expression in spikes collected at different stages of development, for instance, led to an estimation of its transcription level about 2–6 times higher than those obtained using the most stable genes indicated by geNorm and NormFinder (Fig. 5). The misleading effect of using unsuitable reference genes for the normalization of expression data was even more evident when the analysis was performed on the data sets relative to single groups of tissues (vegetative or floral organs, Figs. 6A and 6B) or temperature treatments (Fig. 6C). In the data set including only the vegetative tissues normalization with Ta54825 (Actin) estimated a significantly lower expression level of TaPDIL1-1 (approximately 1.3–3.6 times) in four of the six tissues analysed (Shoots1, Shoots3, Shoots4 and leaves) (Fig. 6A). Moreover, the normalization procedure with Ta25534 (α-tubulin) showed a weak but significant enhancement of TaPDIL1-1 mRNA expression in shoots collected from plants at three leaves unfolded stage, at the beginning of tillering and with formed tillers (Shoots1-3 in Fig. 6A). Among the floral organs, the use of Ta54825 (Actin) and Ta25534 (α-tubulin) as single reference genes led to significant over-estimation of TaPDIL1-1 expression in lodicules and pistil (Fig. 6B). Moreover, when TaPDIL1-1 expression was normalized with Ta54825 (Actin) in stamens its transcription level was about 3.7 times higher than the estimation obtained using the best genes indicated by geNorm and NormFinder (Fig. 6B). Finally, when the normalization of the TaPDIL1-1 gene was carried out using the geometric mean of the reference genes selected by NormFinder and by geNorm, the single best gene identified by NormFinder and Ta54825 (Actin), the comparison of seedlings exposed for 48 h at low (4°C) and high (33°C) temperatures with the control samples exposed to 18°C detected a significant up-regulation of TaPDIL1-1 mRNA (Fig. 6C). It is noteworthy that Ta54825 (Actin) was one of the two most stable genes selected by geNorm in the temperature treatments (Table 3). On the contrary, the normalization of TaPDIL1-1 expression data using Ta25534 (α-tubulin) as reference gene resulted in a significant decrement of its transcription level after 24 and 48 h exposure to low and high temperatures (Fig. 6C). These contrasting results can be explained by the significant up-regulation of Ta25534 (α-tubulin) induced by the cold and heat shocks.

## Discussion

To the best of our knowledge, the present research represents the first effort aimed to the identification and systematic comparison of a large number of potential reference genes and of the corresponding primer pairs specifically designed for gene expression studies in wheat, in particular for qRT-PCR analyses. As mentioned in the "Introduction", so far most studies on gene expression in wheat have been carried out using single reference genes, without any preliminary validation of their expression stability (Additional file 1). The limited information on gene expression publicly available in wheat datasets [27-32] does not allow the strategy followed, for instance, in *Arabidopsis *to identify new reference genes, which has been based on the data furnished by the microarray datasets [12]. For selecting candidate reference genes we have developed an alternative and simple *in silico *method exploiting UniGene and TIGR, two publicly available databases of wheat sequences enclosing non-redundant EST-indices. The method consists of two steps, each exploiting the peculiar features of these wheat-specific databases. Basically, an effective reference gene has to fulfil three main requirements: 1) moderate to high level of expression (above the basal cell background); 2) expression in several tissues and developmental/physiological states; 3) low variation of gene expression across different tissues and developmental, physiological and environmental conditions [15]. These requirements were translated into three stringent criteria imposed for identifying wheat candidate reference genes from *T. aestivum *UniGene database (see Methods), which were accomplished by 177 out of the 42,256 sequence clusters therein contained. The second step consisted in the scanning of the TIGR wheat gene index database (TaGI version 11) for the identification of the TC (Tentative Consensus) or group of TCs corresponding to each of the 177 clusters previously selected in UniGene. The identification of TCs was important because these outputs are more effective for gene functional annotation and for designing gene specific qRT-PCR primers. The frequency (number of hosting libraries/total number of cDNA libraries) of each TC or group of TCs linked to the 177 selected UniGene clusters was determined and was used to pinpoint the most suitable candidate reference genes to be included in a preliminary screening.

Among the 177 *in silico *identified sequences, specific and efficient primers were developed for 32 genes, whose expression level was assessed by qRT-PCR using a set of cDNAs from 24 plant samples, which included different tissues, developmental stages and temperature stresses. The 32 selected sequences consisted of 12 well-known HKGs representing distinct functional classes and 20 genes that either were not related to any housekeeping activity or had unknown functions, therefore they can be assumed as novel in relation to the normalization issue. The UniGene clusters Ta54825, Ta25534, Ta2291 and Ta53964 were included among the 32 selected candidate reference genes because preliminary qRT-PCR analyses showed that they were the most stable of the genes belonging to four analysed gene families: actin, α-tubulin, ADP-ribosylation factor and translation elongation factor, respectively. The simultaneous amplification of two or more reference genes of the same family by a single primer pair designed in the conserved regions has been suggested by some Authors as a strategy for reducing the cost of using multiple and independent control genes [16,20,52]. This approach assumes the balanced expression of different amplified genes of the same family, but this is not always true. For example, *UBQ5 *(*UBIQUITIN 5*) has been one of the most suitable reference genes in a set of tissue samples in rice, whereas the expression of *UBQ10 *was unstable [19]. A similar situation has been observed for the actin gene family in a set of samples collected at different development stages of soybean, where *ACT2/7 *was stably expressed, whereas *ACT11 *showed variable profiling [53]. In wheat and in our experimental conditions the approach based on the use of conserved primer pairs amplifying multiple gene sequences was not successful, in fact the primer pairs amplifying the single best gene for each of the four analysed families performed better than those targeting multiple genes (Table 3).

Since the search for suitable reference genes is both time-consuming and cost intensive, several computer programs based on different statistical approaches have been developed to assist the selection of a reference gene among several HKGs. The software geNorm has been reported in 2002 [13] and has been followed by NormFinder in 2004 [15], they have been very successful and widely used. Also BestKeeper is a freely available program that determines the best-suited reference gene by pair-wise correlation analysis of raw C_t _values [14]. According to Lyng et al. [54], this approach may be useful to narrow down a search if no specific genes are known to be plausible candidates, whereas more advanced statistics, as those provided by geNorm and NormFinder, are needed to rank the genes if several of them are identified as good candidates; moreover BestKeeper can analyse a maximum of ten reference genes. Some more statistical models have been developed for identifying optimal reference genes, but either they are not freely available, their application is complex [55,56], or they do not take the PCR efficiency into account [16]. The choice of the program using the most suitable statistical method represents a crucial but difficult task, because each algorithm has the potential of delivering different results, as demonstrated by several studies [20,53,57,58]. Since there is no universally and univocally accepted method to rank candidate reference genes on the basis of their expression stability, the present study analysed a set of wheat samples using GeNorm [13] and NormFinder [15] and compared the results obtained by the two programs. This comparison showed some discrepancies in the ranking of the candidate reference genes and in the identification of the best ones obtained by the two programs, but there was substantial agreement if the grouping of the genes with the most and least stable expression was considered (Table 5).

As discussed by different authors [15,57-59], since geNorm determines the reference gene stability by pair-wise comparison of the variation of expression ratios, it needs a careful choice of the candidate genes included in the analysis. In particular, the candidate genes should not be co-regulated to avoid the selection of genes with the highest similarity of expression pattern but not stably expressed, consequently not suitable as reference genes. To reduce the chance of using co-regulated genes, in our study special attention was paid in order to select for qRT-PCR analysis genes involved in distinct biological processes and metabolic pathways, even though for some of the new reference genes selected (e.g. Ta54733, Ta54448, Ta53889 and Ta53891 in Table 2) the functional information associated with their annotation was inadequate. Moreover, it should be remarked that besides similarities of gene expression pathways other factors can contribute to co-regulation. Yu et al. [60], for instance, showed that genes targeted by similar transcription factors have complex relationships across the co-regulated genes. Since some studies have shown that the removal of genes from the analysis modified the ranking of the candidate genes [58,61], we checked for possible co-regulation between the most stably expressed genes by removing single high ranking genes (as determined by pair wise comparisons), and reassessing the rank ordering of the remaining reference genes. Except for the floral organs (IV Data set), changes in rank ordering of the ten most stable genes were observed in the remaining four data sets when single high ranking genes were removed from geNorm analysis (data not shown).

The mathematical model of gene expression used by NormFinder enables to estimate both the overall variation of the candidate normalization genes and the variation between subgroups of the sample set. This approach has been reported to perform in a more robust manner than geNorm and has been shown to be less sensitive to the presence of co-regulated genes [15], therefore the choice of candidate reference genes without functional relationships is less critical than in geNorm. A very useful information given by geNorm is the ideal number of reference genes which should be included in a NF (Normalization Factor), whereas NormFinder indicates only the best single gene and the best combination of two genes. As shown by several studies [15,62,63], the normalization based on multiple reference genes gives more accurate evaluation of gene expression, especially when no single optimal reference gene is available. The cost/benefit evaluation is an important issue in the decision on the number of reference genes to be used for normalization, in particular when large experiments, based on the analysis of a number of tissues, are planned. In such cases a compromise needs to be achieved between the instances of economical adequacy and the required degree of accuracy. In all the four data sets analysed to study the expression of TaPDIL-1-1, we observed that the normalization obtained using the single most stable reference gene identified by NormFinder was comparable to that based on the NF computed using the geometric mean of the two or three most stable genes selected by NormFinder and geNorm, respectively (Figs. 5 and 6). It might be concluded that in our experimental conditions a single reference gene was sufficient to obtain an adequate normalization of the qRT-PCR data and that NormFinder was more effective than geNorm in selecting the most effective reference genes.

Expression stability analyses showed that the most stable reference gene identified by NormFinder and geNorm was not the same for the five data sets analysed (Table 5). Even though our data confirm, as shown by most papers on the subject, that it is not possible to find an ideal all purpose reference gene, they highlight the convenience of pilot studies to select the most suitable gene for specific experimental conditions, as shown also by Gutierrez et al. [23]. When the complete data set including 24 samples was analysed, NormFinder identified Ta54227 (Cell division control prot., AAA-superfamily of ATPases), Ta2291 (ADP-ribosylation factor) and Ta2776 (RNase L inhibitor-like protein) as the most stably expressed genes (Table 4), whereas in geNorm the same genes ranked among the seven most stable genes (Table 5). Ta54227 can be identified as the best reference gene to normalize expression data from different wheat tissues and developmental stages, in fact NormFinder included Ta54227 among the two most stable genes in three of the analysed data sets (II, IV and V data sets, Table 4). Also geNorm included Ta54227 among the four most stable genes in three of the analysed data sets (Figs A, C and D in Additional file 11). However, Ta54227 is not suitable as control gene for analysing the samples subjected to cold and heat shocks, because there was considerable variation of its expression in this data set, wherein Ta2291 and Ta2776 performed much better (Table 5). Moreover, since the expression level of Ta2776 is low (mean C_t _24.79), whereas that of Ta2291 (mean C_t _21.97) and of Ta54227 (mean C_t _21.56) is high, they are suitable as reference genes in a large data set of wheat samples, but also for the normalization of target genes with contrasting expression levels.

It is noteworthy that most of the novel reference genes identified in this study were more stably expressed than well-known and frequently used HKGs, such as Actin (Ta54825), α-tubulin (Ta25534), β-tubulin (Ta44405), Ubiquitin (Ta50503), GAPDH (Ta30768), Cyclophilin (Ta35497), Translation elongation factor (Ta53964), Translation initiation factor (Ta54280), Ribosomal protein (Ta27771) and Histone (Ta38797), in fact most of them classified as the least stable genes both by geNorm and NormFinder in the five data sets analysed (Table 5). When the complete data set including 24 samples was analysed by NormFinder, Ta22845 (ATP-dependent 26S proteasome regulatory subunit), which resulted the gene with the highest expression stability among the selected traditional HKGs, ranked only 12^th ^among the 32 candidate reference genes (I Data set in Table 4). In the 18 samples including different tissues/organs at different developmental stages of wheat the best HKG identified by NormFinder was Ta35497 (Cyclophilin) which ranked only tenth (II Data set in Table 4), whereas in the temperature treatment series the most stable HKG was again Ta22845 (position 8^th ^in III Data set of Table 4). Finally, Ta44405 (β-tubulin) was identified as the most stable HKG among the single floral organs (position 10 in the IV Data set of Table 5), whereas only in the six samples including vegetative tissues Ta50503 (ubiquitin), a traditional HKG, was classified as one of the most stable genes both by NormFinder and geNorm (V data set in Table 5).

The example of expression level analysis of TaPDIL1-1 shows that the selection of stable reference genes represents a crucial issue for the correct normalization of qRT-PCR data. The comparison of the relative TaPDIL1-1 mRNA expression using different normalization approaches (Figs. 5 and 6) showed that Actin (Ta54825) and α-tubulin (Ta25534), most frequently used in wheat gene profiling studies (Additional file 1), and most traditional HKGs (data not shown) analysed in our research were less effective than the best reference genes identified in this study.

## Conclusion

In the present research we compared 20 novel candidate reference genes and 12 commonly used HKGs suitable for gene expression normalization in wheat. Both new and traditional reference gene sequences were fetched by an *in silico *cross search for stable expression in Unigene and TIGR databases. The expression stability of the 32 selected genes was assessed by qRT-PCR in 24 different plant samples, which included different tissues, developmental stages and plants exposed to temperature stresses. The computer programs geNorm and NormFinder were used to compare the expression patterns of the 32 candidate genes and to identify the best reference genes, which were not the same. However, when single genes were not considered, compatible results and substantial agreement was observed between the two programs in discriminating between groups with the most and least stable expression. Our results indicate that many of the new identified reference genes outperform the traditional HKGs in terms of expression stability under all the tested conditions. Ta54227 (Cell division control prot., AAA-superfamily of ATPases), Ta2291 (ADP-ribosylation factor) and Ta2776 (RNase L inhibitor-like protein) resulted the best and most stable reference genes to normalize gene expression in different tissue and development stages of wheat; they outperformed all traditional HKGs such as α-tubulin (Ta25534), β-tubulin (Ta44405), Ubiquitin (Ta50503), Actin (Ta54825), GAPDH (Ta30768), Ribosomal protein (Ta27771) and Histone (Ta38797). Although in our experimental conditions a single reference gene was adequate to normalize the qRT-PCR data, the use of normalization factors generated from the expression data of two or three most stable genes should be considered in future studies to improve the accuracy and reliability of expression analysis. The set of qRT-PCR specific primers for the novel and more stable reference genes developed in this study will enable more accurate normalization and quantification of gene expression in wheat. Moreover, the design of suitable primer pairs for their orthologous genes could contribute to the development of reference genes with stable expression in other plant species.

## Abbreviations

qRT-PCR: quantitative Real-time RT-PCR; HKG: Housekeeping gene; GAPDH: glyceraldehyde-3-phosphate dehydrogenase; RT: Reverse transcriptase; EST: expressed sequence tag; TIGR: The Institute for Genome Research; PDI: Protein disulphide isomerase; TPM: transcripts per million; TC: tentative consensus sequence; Mv: mean value; V: variance; SD: standard deviation; CV: coefficient of variation; SSH: Suppression Subtractive Hybridization; CS: Chinese Spring; TEF-1α: Translation Elongation Factor-1α; ADP-RF: ADP-ribosylation factor; GO: Gene Ontology; ANOVA: analysis of variance; MST: mean square of treatments; MSE: mean square errors; NF: normalization factor; NCBI: National Center for Biotechnology Information.

## Authors' contributions

ARP performed all the sample preparations and experimental procedures and participated in data analysis. MC supervised the study, performed data analysis and draft the manuscript. OAT performed data analysis, revised the manuscript critically and participated in tables and figures drawing. EP revised critically the manuscript and gave financial support to the study. All authors read and approved the final manuscript.

## Supplementary Material

Additional file 1 **List of articles reporting the application of reference genes for qRT-PCR normalization in wheat.** The list was obtained by a PubMed search from January 1996 to March 2008 and includes 26 articles reporting 16 reference genes. Click here for file

Additional file 2 **RT-PCR primers used in this research.** Table showing UniGene clusters and linked TC sequences of the reference genes used in qRT-PCR analysis, their primer sequences and characteristics of the corresponding amplicons. Click here for file

Additional file 3 **UniGene clusters and corresponding TC sequences of 177 selected candidate reference genes of wheat.** The file reports the transcription profiles of the genes corresponding to the UniGene clusters in six wheat tissues (flower, inflorescence, leaf, root, seed and stem) calculated as a value representing the number of transcripts per million (TPM). Mean value (Mv), Variance (V), Standard deviation (SD) and Coefficient of Variation (CV) were determined on the expression values (TPM) of the 177 Unigene clusters in the six wheat tissues. The highest and lowest expression values, the relative expression (percentage) in the six wheat tissues and the number of ESTs included in each UniGene cluster are also reported. The frequency (number of hosting libraries/total number of cDNA libraries) was determined for each TC or group of TCs linked to the 177 selected UniGene clusters. Click here for file

Additional file 4 **Classification of the 177 selected candidate reference genes of wheat on the basis of their putative functions.** File showing the UniGene clusters and corresponding TC sequences of the 177 selected candidate reference genes of wheat and their putative functional annotation determined on the basis of their orthologous genes of rice and relative GO annotations. Click here for file

Additional file 5 **Level of expression of the 177 selected candidate reference genes determined on the basis of the number of EST sequences included in each UniGene cluster.** File showing UniGene clusters and corresponding TC sequences of the 177 selected candidate reference genes of wheat sorted in ascending order on the basis of the number of EST sequences included in each UniGene cluster. Click here for file

Additional file 6 **UniGene clusters and corresponding TC sequences of ten genes of the actin and α-tubulin families.** Gene transcription profile of each UniGene cluster in six wheat tissues (flower, inflorescence, leaf, root, seed and stem) is reported as a value representing the number of transcripts per million (TPM). Mean value (Mv), Variance (V), Standard deviation (SD) and Coefficient of Variation (CV) were calculated on the expression values (TPM) of the ten Unigene clusters in the six wheat tissues. The highest and lowest expression values, the relative expression (percentage) in the six wheat tissues and the number of ESTs included in each UniGene cluster are also reported. The frequency (number of hosting libraries/total number of cDNA libraries) was determined for each TC or group of TCs linked to the 177 selected UniGene clusters. Click here for file

Additional file 7 **Expression analysis by RT-PCR of the genes *wcor14 *and *TaHSP101B*.** Agarose gels of RT-PCR products of *wcor14 *and *TaHSP101B *genes after 35 PCR cycles in six samples consisting of two temperature treatments (4°C and 33°C) for 24 and 48 h and their controls (LTC = low temperature controls; HTC = high temperature controls). The transcripts of the constitutively expressed gene encoding actin (UniGene cluster Ta54825) were amplified as control. M = part of the DNA molecular weight marker XIV (Roche), the most intense band is 500 bp in length. Click here for file

Additional file 8 **Specificity of qRT-PCR amplification.** Figures A1–A3 report the dissociation curves of 42 selected reference genes showing single peaks, they were obtained from three technical replicates of 18 cDNA pools representing different tissues and developmental stages of wheat. Figures B1–B3: Agarose gels (2%) showing amplification of a specific PCR product of the expected size for each candidate reference gene. Click here for file

Additional file 9 **PCR efficiency and C_t _values for 46 primer pairs and 24 tissue samples used in this research.** The file shows PCR efficiency and C_t _values of two biological replicates performed on 24 tissue samples; three technical replicates were carried out for each of the 46 primer pairs. The 24 tissue sample comprised: 1) Roots from plants with single shoot and three leaves unfolded (Feekes scale 1.3); 2) Shoots1 = single shoots and leaves from plants at Feekes scale 1.3; 3) Shoots2 = shoots at the beginning of tillering (Feekes scale 2); 4) Shoots3 = shoots from plants with formed tillers (Feekes scale 3); 5) Shoots4 = shoots at the beginning of erect growth (Feekes scale 4); 6) flag leaves at booting stage (Feekes scale 10); 7) stems at booting stage (Feekes scale 10); 8–10) Spikes1–3 = spikes collected at intervals of 10–12 days (three developmental stages: 15–20 mm, flag leaf unfolding and heading stage); 11–16) single floral organs (glumes, lemma, palea, lodicules, stamens and pistil) from fully emerged spikes (Feekes scale 10.5); 17–18) Seeds 1–2 = seeds collected 15 (medium milk stage) and 30 (hard dough stage) days after anthesis; 18–24) six samples (seedlings at Feekes scale 1.3) consisting of two temperature treatments (4°C and 33°C) for 24 and 48 h and their controls (LT = low temperature; HT = high temperature). Click here for file

Additional file 10 **Expression stability of the genes belonging to four families (actin, α-tubulin, translation elongation and ADP-ribosylation factors) evaluated by statistical analysis of raw C_t _values obtained in five different data-set. **ANOVA analysis and statistics determined on raw C_t _values obtained by 18 primer pairs which amplified either specific or multiple (m) gene transcripts of four gene families (Actin, α-tubulin, translation elongation and ADP-ribosylation factors) in five different data-sets: 1) all 24 samples including tissues, developmental stages and temperature treatments; 2) 18 samples relative to different tissues and developmental stages; 3) six samples consisting of two temperature treatments (4°C and 33°C), each for 24 and 48 h and their controls; 4) six samples relative to the single floral organs from fully emerged spikes; 5) six samples referring to vegetative tissues and developmental stages (shoots, stems and leaves). Click here for file

Additional file 11 **Ranking of the expression stability of the reference genes as calculated by geNorm in four data sets.** geNorm output charts of M values for the 32 selected reference genes in four data sets: (A) = 18 tissues and developmental stages; (B) = six samples consisting of two temperature treatments (4°C and 33°C) for 24 and 48 h and their controls; (C) = six floral organs from fully emerged spikes; (D) = six vegetative tissues and developmental stages (shoots, stems and leaves). Click here for file

Additional file 12 **Determination of the optimal number of control genes for accurate normalization calculated on the basis of pair-wise variation (V) analysis in four data sets.** geNorm output charts of V values for the 32 selected reference genes in four data sets: (A) = 18 tissues and developmental stages; (B) = six samples consisting of two temperature treatments (4°C and 33°C) for 24 and 48 h and their controls; (C) = six floral organs from fully emerged spikes; (D) = six vegetative tissues and developmental stages (shoots, stems and leaves). Click here for file
