# Supplementary material for: Identification and validation of reference genes for quantitative RT-PCR normalization in wheat
Source: BMC Mol Biol. 2009 Feb 20;10:11. doi: 10.1186/1471-2199-10-11 (PMC2667184; doi:10.1186/1471-2199-10-11)
Supplement: Additional file 1 — List of articles reporting the application of reference genes for qRT-PCR normalization in wheat. The list was obtained by a PubMed search from January 1996 to March 2008 and includes 26 articles reporting 16 reference genes. [file 1471-2199-10-11-S1.doc]

# Paper Reference gene Primer Validation Tissues Use of real time RT-PCR

**1**

Kong, Lingrang; Ohm, Herbert W.; Anderson, **18S rRNA** **(AY049040) Yes No Glumes Expression analysis of 51 cDNA**

Joseph M. **Expression analysis of defense fragments differentiallyexpressed**

**related gene s in wheat in response to (GeneCalling Technology)**

**infection by Fusarium graminearum.**

Genome **(2007**) 50(11) 1038-1048

**2**

Yu, Xiu-Mei; Yu, Xiu-Dao; Qu, Zhi-Peng; **18S rRNA** **(AY049040) Yes No Leaves Expression analysis of a HIR gene**

Huang, Xin-Jie; Guo, Jun; Han, Qing-Mei; ***Ta-hir1***

Zhao, Jie; Huang, Li-Li; Kang, Zhen-Sheng.

**Cloning of a putative hypersensitive induced**

**reaction gene from wheat infected by tripe**

**rust fungus.** Gene **(2008)** 407(1-2), 193-198.

**3**

Liu, Yong; Liu, Dongcheng; Zhang, Haiying;

Gao, Hongbo; Guo, Xiaoli; Wang, Daowen;

Zhang, Xiangqi, Zhang, Aimin.

**The α - and β -expansin and xyloglucan** **Alpha-tubulin gene Yes No Different tissues Expression analysis of five**

**endotransglucosylase/hydrolase gene Nodes, Internodes wheat members of a and b**

**families of wheat : Molecular cloning, gene Leaves, Spikes EXP and XTH gene families**

**expression, and EST data mining. 10 days old seedlings**

Genomics **(2007)** 90(4) 516-529 **treated with hormones**

**4**

Cazalis, Roland; Pulido, Pablo; Aussenac, **18S rRNA** **(AY049040) Yes No Different tissues: Expression analysis of**

Thierry; Perez-Ruiz, Juan Manuel; Cejudo, **seeds collected at 3 Trxh genes**

Francisco Javier. **Cloning and characterization different stages of**

**of three thioredoxin h isoforms from wheat development, spikes**

**showing differential expression in seeds. after ripening, leaves,**

Journal of Experimental Botany **seedlings in stress**

**(2006)**, 57(10), 2165-2172. **conditions (NaCl, H2O2**)

**5**

Vagujfalvi, Attila; Aprile, Alessio; Miller, **Actin gene** **Yes No Seedlings exposed Expression analysis of**

Andrea; Dubcovsky, Jorge; Delugu, **to low temperatures 8 Cbf genes**

Giovanni; Galiba, Gabor; Cattivelli, Luigi **(2 °C)**

**The expression of several Cbf genes at the**

**Fr-A2 locus is linked to frost resistance in**

**wheat.** Molecular Genetics and Genomics

**(2005)**, 274(5), 506-514.

# Paper Reference gene Primer Validation Tissues Use of real time RT-PCR

**6**

Kawaura Kanako; Mochida Keiichi; Yamazaki **Polyubiquitin gene Yes The expression Seedlings two weeks Validation of microarray**

Yukiko; Ogihara Yasunari **of the gene in old treated with NaCl analysis (12 genes)**

## Transcriptome analysis of salinity stress the microarray

**responses in common wheat using a analysis was**

**22k oligo-DNA microarray**. u**nchanged by**

Functional & integrative genomics **(2006)**, **salt treatment**

6(2), 132-42.

**7**

Ali-Benali, Mohamed A.; Alary, Remi; **26S rRNA** **(M37274) Yes No Seeds collected at Expression analysis of**

Joudrier, Philippe; Gautier, Marie-Francoise. **various stages of 5 Lea genes**

**Comparative expression of five Lea Genes development, coleoptiles**

**during wheat seed development and and roots from seedlings**

**in response to abiotic stresses by real time one week old exposed to**

**quantitative RT - PCR.** Biochimica et **dehydratation, low T**,

Biophysica Acta, Gene Structure and **salinity and ABA**

Expression **(2005)**, 1730(1), 56-65.

**8**

Kong, Lingrang; Anderson, Joseph M.; **18S rRNA** **(AY049040) Yes No Glumes Validation of 6 cDNA**

Ohm, Herbert W.  **Induction of wheat clones differentially**

**defense and stress-related genes in expressed and identified**

**response to Fusarium graminearum. by SSH tecnique**

Genome **(2005)**, 48(1), 29-40.

**9**

Amoroso, M. G.; Longobardo, L.; Capparelli, R **18S rRNA** **(AY049040) Yes No Seeds collected at Expression analysis of**

**Real Time RT - PCR and flow cytometry various stages of 2 puroindoline genes**

**to investigate wheat kernel hardness: development**

**role of puroindoline genes and proteins.**

Biotechnology Letters **(2004)**,

26(22), 1731-1737.

**Paper Reference gene Primer Validation Tissues Use of real time RT-PCR**

**10**

Ray, Suparna; Anderson, Joseph M.; Urmeev, **18S rRNA** **(AY049040) Yes No** **Seedlings at** **Expression analysis of**

Flora I.; Goodwin, Stephen B. **5-6 leaf stage a PDI gene and of 5**

**Rapid induction of a protein disulfide inoculated with defense-related genes**

**isomerase and defense-related genes in wheat a fungal pathogen (PR and LOX)**

**in response to the hemibiotrophic fungal**

**Mycosphaerella graminicola.**

Plant Molecular Biology (2003), 53(5),

701-714.

**11**

Wan Yongfang; Poole Rebecca L; Huttly **Gene coding for a DSS1/ No The transcript Seeds collected Validation of microarray**

Alison K; Toscano-Underwood Claudia; **SEM1 proteasome relative to the at various stages analysis (9 genes coding for**

Feeney Kevin; Welham Sue; Gooding Mike J; **subunit** **family protein probeset** **of development** **transcription factors)**

Mills Clare; Edwards Keith J; Shewry Peter R; **(Ta.2526.1.S1_at) Ta.2526.1.S1_at**

Mitchell Rowan Ac. **Transcriptome analysis shows constant**

**of grain development in hexaploid wheat**. **expression in array**

BMC genomics **(2008)**, 9 121 **data for all samples**

**analysed**

**12**

Mott, Ivan W.; Wang, Richard R.-C. **Gene coding for a Yes No Shoots and roots Validation of microarray**

**Comparative transcriptome analysis translational elongation from plants under analysis (2 genes)**

**of salt-tolerant wheat germplasm lines factor EF1a salt stress conditions**

**using wheat genome arrays. (AFFX-Ta-ef1 a-M_s_at) (NaCl)**

Plant Science (Amsterdam, Netherlands)

**(2007**), 173(3), 327-339.

**13**

Peiguo, Guo; Guihua, Bai; Brett, Carver; **Gene coding for a 40S No The expression Roots from four Validation of microarray**

Ronghua, Li; Amy, Bernardo; Baum, Michael. **ribosomal protein S8 of the 40S gene day old seedlings analysis (ten genes)**

**Transcriptional analysis between two wheat was similar under Al stress**

**Near-isogenic lines contrasting in aluminium during various**

**Tolerance under aluminium stress. time of Al stress**

Molecular Genetics and Genomics **(2007)** 277: **in all samples**

1-12 **analysed**

**Paper Reference gene Primer Validation Tissues Use of real time RT-PCR**

**14**

Crismani, Wayne; Baumann, Ute; Sutton, **Six different genes:** **Yes** **The three best Anthers analysing Validation of microarray**

Tim; Shirley, Neil; Webster, Tracie; Spangenberg, **Actin, Cyclophilin control genes seven different analysis (15 genes)**

German; Langridge, Peter; Able, Jason A **EFAI, GAPDH, from the set stages of meiosis**

**Microarray expression analysis of meiosis Ta.9657.I.SI_at, of the six**

**and microsporogenesis in hexaploid bread Ta.28350.I.SI_a_at analysed were**

**wheat.** BMC Genomics (2006), **selected using**

**geNorm**

**15**

Xue, Gang-Ping; McIntyre, C. Lynne; **Gene coding for Yes The expression Mature leaves Validation of microarray**

Chapman, Scott; Bower, Neil I.; Way, Heather; **TaRPII36 a** **RNA of the gene was from field grown analysis (22 genes)**

Reverter, Antonio; Clarke, Bryan; Shorter, Ray. **polymerase II similar in the and drought stressed**

**Differential gene expression of wheat progeny 36 KDa subunit samples from plants of two**

**with contrasting levels of transpiration efficiency. (TC235230) the wheat wheat cvs and**

Plant Molecular Biology **(2006)**, 61(6), 863-881. **genotypes** **progenies derived**

**analysed from a cross**

**between the**

**same two cvs**

**16**

Gregersen, Per L.; Brinch-Pedersen, Henrik; **18S rRNA** **(AY049040) No No Seeds collected Validation of microarray**

Holm, Preben B. **A microarray-based at various stages analysis (7 genes)**

**comparative analysis of gene expression of development**

**profiles during grain development in**

**transgenic and wild type wheat.**

Transgenic Research **(2005)**, 14(6), 887-905.

**17**

Clarke, Bryan; Rahman, Sadequr. **Alpha-tubulin gene Yes The gene was Seeds collected Validation of microarray**

**A microarray analysis of wheat grain selected from at various stages analysis (4 genes)**

**hardness.** Theoretical and Applied Genetics **the microarray of development**

**(2005)**, 110(7), 1259-1267. **data on the basis**

**of its stable**

**expression**

**18**

Bernardo Amy; Bai Guihua; Guo Peiguo; **Actin gene Yes The expression Spikes at anthesis Validation of microarray**

Xiao Kai; Guenzi Arron C; Ayoubi Patricia **of the gene inoculated with analysis (8 genes)**

**Fusarium graminearum-induced changes in was uniform F. graminearum**

**gene expression between Fusarium head blight- across all**

**resistant and susceptible wheat cultivars**. **samples**

Functional & integrative genomics **(2007**), **analysed**

7(1), 69-77.

**Paper Reference gene Primer Validation Tissues Use of real time RT-PCR**

**19**

Lu Chungui; Hawkesford Malcolm J; **Actin gene (AL825219) No No Seeds 14 dpa Validation of microarray**

Barraclough Peter B; Poulton Paul R; **Alpha tubulin from plants analysis (12 genes)**

Wilson Ian D; Barker Gary L; **gene (AL825219) subjected to**

Edwards Keith J  **Markedly different seven nitrogen**

**gene expression in wheat grown treatments and**

**with organic or inorganic fertilizer**. **growth in different**

Proceedings. Biological sciences / **UK locations**

The Royal Society **(2005)**,

272(1575), 1901-8.

**20**

Xue, Gang-Ping; McIntyre, C. Lynne; Jenkins, **Gene coding for Yes The mRNA Stems from plants Validation of microarray**

Colin L. D.; Glassop, Donna; van Herwaarden, **TaRPII36 a** **RNA level of the at the heading of analysis (15 genes)**

Anthony F.; Shorter, Ray. **Molecular dissection polymerase II gene was stable 16 recombinant**

**of variation in carbohydrate metabolism 36 KDa subunit among the inbred lines**

**related to water- soluble carbohydrate (TC235230) samples**

**accumulation in stems of wheat.** **analysed**

Plant Physiology **(2008)**, 146(2), 441-454.

**21**

Stephenson, Troy J.; McIntyre, C. Lynne; Collet, **Four genes: Yes TaRPII36 was Different tissues: Expression analysis of**

Christopher; Xue, Gang-Ping. **Genome-wide TaRPII36 (TC235230) used as referen- roots, leaves, stems, 34 gene of the NF-Y**

**identification and expression analysis of the TaRP15 (TC26512) ce gene because spikes from plants transcription factor family**

**NF-Y family of transcription factors in TaCCF (BJ309282) of its lower at the pre-anthesis**

**Triticum aestivum.** Plant Molecular **TaPGM2 (TC263735) variability stage, endosperm**

Biology **(2007**), 65(1-2), 77-92 **among the and embryo from**

**analysed seeds 20-30 dpa;**

**samples leaves from drought**

**stressed plants**

**22**

Badawi, Mohamed; Danyluk, Jean; Boucho, **18S rRNA** **(AY049040) No No Seedlings growth at Expression analysis of**

Barbara; Houde, Mario; Sarhan, Fathey. l**ow temperature CBF gene family (13 genes)**

**The CBF gene family in hexaploid wheat**

**and its relationship to the phylogenetic**

**complexity of cereal CBFs.**

Molecular Genetics and Genomics **(2007**),

277(5), 533-554.

**Paper Reference gene Primer Validation Tissues Use of real time RT-PCR**

**23**

Gulli, Mariolina; Corradi, Massimiliano; **Alpha-tubulin gene** **Yes** **No** **Seedlings exposed to Expression analysis of**

Rampino, Patrizia; Marmiroli, Nelson; **(U76558) high temperatures four members of the**

Perrotta, Carla. **Four members of the HSP101 gene family**

**HSP101 gene family are differently**

**regulated in Triticum durum Desf.**

FEBS Letters **(2007)**, 581(25), 4841-4849.

**24**

Shitsukawa, Naoki; Tahira, Chikako; Kassai, **Actin gene Yes No Spikes at 8 stages of Expression analysis**

Ken-ichiro; Hirabayashi, Chizuru; Shimizu, **(AB181991) development. of three homoeologous**

Kawaura, Kanako; Ogihara, Yasunari; Murai, Koji. **Floral organs: genes of a class E of the MADS**

Tomoaki; Takumi, Shigeo; Mochida, Keiichi; **glumes, lemmas, ,** **box genes**

**Genetic and epigenetic alteration among three paleas stamens,**

**homoeologous genes of a class E MADS box gene pistils**

**in hexaploid wheat.** Plant Cell  **(2007)**, 19(6),

1723-1737.

**25**

Paolacci Anna Rita; Tanzarella Oronzo A; **Actin gene Yes No Spikes at 6 Expression analysis of six**

Porceddu Enrico; Varotto Serena; **(AY663392) development stages. SEPALLATA-like genes**

Ciaffi Mario **Molecular and phylogenetic Floral organs : glumes, of the MADS box transcription**

**analysis of MADS-box genes of MIKC type lemmas, paleas, factor family**

**and chromosome location of SEP-like lodicules, stamens, pistils.**

**genes in wheat ( Triticum aestivum L.)**. **Developing seeds between**

Molecular genetics and genomics : MGG **(2007)**, **0 and 40 dpa (8 samples)**

278(6), 689-708.

**26**

Xue, Gang-Ping; Bower, Neil I.; McIntyre, **Four genes: No The TaCCF Roots and leaves Expression analysis of**

C. Lynne; Riding, George A.; Kazan, **Actin, tubulin genes was from 7-8 week a gene (TaNAC69) of the**

Kemal; Shorter, Ray. **TaNAC69 from the TaRPII36 selected for old plants subjected NAC family of**

**NAC superfamily of transcription factors (TC235230) normalization to drought and ABA transcription factors**

**wheat and recognises two consensus DNA TaCCF because of treatments**

**binding sequences.** Functional Plant Biology **(BJ309282)** **its lower**

**(2006)**, 33(1), 43-57 **variability**
