# Supplementary material for: Identification and validation of reference genes for quantitative RT-PCR normalization in wheat
Source: BMC Mol Biol. 2009 Feb 20;10:11. doi: 10.1186/1471-2199-10-11 (PMC2667184; doi:10.1186/1471-2199-10-11)
Supplement: Additional file 2 — RT-PCR primers used in this research. Table showing UniGene clusters and linked TC sequences of the reference genes used in qRT-PCR analysis, their primer sequences and characteristics of the corresponding amplicons. [file 1471-2199-10-11-S2.doc]

| **UniGene**  **cluster** | **TIGR contig (TC)** | **Amplicon**  **pb** | **Amplicon**  **Tm (°C)** | **Foward primer** | **Reverse primer** |
| --- | --- | --- | --- | --- | --- |
| **Ta4344** | **TC289397** | **178** | **82.08** | **5’-GTCTGGATTGGAGGGTCTATC-3’** | **5’-AACCACCACCTAATAATGCTAC-3’** |
| **Ta54825** | **TC295670, TC296528** | **215** | **82.80** | **5’-TGACCGTATGAGCAAGGAG-3’** | **5’-CCAGACAACTCGCAACTTAG-3’** |
| **Ta53908** | **TC300214, TC294591** | **280** | **82.80** | **5’-CGGTATCGTAAGCAACTG-3’** | **5’-GCGTATCCTTCGTAAATGG-3’** |
| **Ta1868** | **TC300683** | **109** | **81.30** | **5’-AGCACTTTCCAGCAGATG-3’** | **5’-CCCTTTGTAAACAACCTTCC-3’** |
| **Ta20863** | **TC302751** | **117** | **82.10** | **5’-TCAGCACATTCCAGCAGATG-3’** | **5’-GCAGAGAGACACGGAGTAAG-3’** |
| **Actin (m)** |  | **206** | **82.60** | **5’-GAATCCATGAGACCACCTAC-3’** | **5’-AATCCAGACACTGTACTTCC-3’** |
| **Ta5566** | **TC277566, TC277290, TC314308** | **278** | **82.30** | **5’-CTGGGCTCTCTGTTGTTG-3’** | **5’-GAGGTGGTAAGTGAGGATATG-3’** |
| **Ta53981** | **TC365448** | **131** | **83.60** | **5’-ATGTACCGTGGTGATGTT-3’** | **5’-CCTGGTGGCTGGTAGTTG-3’** |
| **Ta33558** | **TC315647, TC285752** | **111** | **83.15** | **5’-CAAGGATGTCAACGCTGCTGTG-3’** | **5’-ACGCTGGGTGGCTGGTAG-3’** |
| **Ta54519** | **TC311662, TC285782** | **235** | **86.20** | **5’-CGTGGACTATGGAAAGAA-3’** | **5’-TGAGATCACCTGAGAGAC-3’** |
| **Ta25534** | **TC280933, TC307866, TC279800** | **150** | **84.65** | **5’-GCCATCTACGACATCTGC-3’** | **5’-GGTCTGGAACTCGGTTATG-3’** |
| **Alpha tubulin (m)** |  | **176** | **84.25** | **5’-CCATCAAGACCAAGCG-3’** | **5’-GTCAATGCGGGAGAAG-3’** |
| **Ta659** | **TC288390** | **97** | **74.90** | **5’-CAAGGGTGTGGAGAAGAAGG-3’** | **5’-AGCAGACATAGATGGATTCAGG-3’** |
| **Ta53964** | **TC279148** | **164** | **77.00** | **5’-CGGTTGCTGTTGGTGTCATC-3’** | **5’-TCATTGCTCGCTCGGGATAAG-3’** |
| **TEF-1α (m)** |  | **233** | **78.30** | **5’-GATTGGTGGCATTGGAAC-3’** | **5’-GGTCATCCTTGGAGTTGG-3’** |
| **Ta45379** | **TC337136** | **140** | **72.70** | **5’-CTGGCTGTCCAACAACATTGC-3’** | **5’-AACACCCACATTATCTGTCAAGAC-3’** |
| **Ta2291** | **TC278558, TC278370** | **165** | **73.70** | **5’-GCTCTCCAACAACATTGCCAAC-3’** | **5’-GCTTCTGCCTGTCACATACGC-3’** |
| **ADP-RF (m)** |  | **276** | **78.70** | **5’-GACCACCATCCTCTACAAG-3’** | **5’-AGCAGCACAGCATCAC-3’** |
| **Ta44405** | **TC278209** | **136** | **77.90** | **5’-GCTTGCTGTCAATCTCATC-3’** | **5’-CTTGGCATCCCACATTTG-3’** |
| **Ta50503** | **TC278495, TC296829, TC280981** | **131** | **77.60** | **5’-GCACCTTGGCGGACTACAACATTC-3’** | **5’-GACACCGAAGACGAGACTTGTGAACC-3’** |
| **Ta30768** | **TC304401, TC348958, TC310664** | **175** | **76.90** | **5’-TTGCTCTGAACGACCATTTC-3’** | **GACACCATCCACATTTATTCTTC-3’** |
| **Ta35497** | **TC348130, TC348051, TC352472** | **131** | **77.00** | **5’-GTGTGTCCCGTGTCGTGTC-3’** | **5’-TCCAGCAGCCCAAAGAGTCC-3’** |
| **Ta38797** | **TC290356, TC280404, TC313660** | **109** | **79.30** | **5’-GTCACCATCATGCCCAAG-3’** | **5’-CAACACATTCCACTTCCG-3’** |
| **Ta54280** | **TC297312, TC338691** | **137** | **74.10** | **5’-TCTGCTGTAATGGTACTGTAGTC-3’** | **5’-TCCGTGAATCTTGATGCTCTC-3’** |
| **Ta54238** | **TC295800, TC289972, TC289612** | **113** | **77.20** | **5’-TTCTTTTCTCACAACCCAACGAC-3’** | **5’-GCCTCCCGACATTGCCATCTG-3’** |
| **Ta27771** | **TC363506, TC298941** | **179** | **80.60** | **5’-CAAGGAGTACCGTGACAC-3’** | **5’-GCGGGAACTTGATCTTCG-3’** |
| **Ta54963** | **TC304353, TC304722, TC295997** | **111** | **75.50** | **5’-AGGAGAACAAGGACGAGGAC-3’** | **5’-AGGAGGCATTCAGAGCGATTG-3’** |
| **Ta54171** | **TC317053, TC294760, TC297148** | **88** | **75.50** | **5’-TGAGCAAGAGCACTGGAAAC-3’** | **5’-CGTTGGTCGGCGAAGATG-3’** |
| **Ta54948** | **TC278352, TC303984** | **161** | **76.60** | **5’-GAACGAGCCAGAGGAAGAAC-3’** | **5’-AAATACGGGTCCACAAGTCAC-3’** |
| **Ta2776** | **TC278756, TC314244** | **242** | **73.50** | **5’-CGATTCAGAGCAGCGTATTGTTG-3’** | **5’-AGTTGGTCGGGTCTCTTCTAAATG-3’** |
| **Ta30797** | **TC279294, TC284282** | **126** | **76.70** | **5’-GCCGTGTCCATGCCAGTG-3’** | **5’-TTAGCCTGAACCACCTGTGC-3’** |
| **Ta35284** | **TC278525** | **224** | **75.70** | **5’-AGCAATTCGCACAATTATTACAAG-3’** | **5’-CTCACAGAAGACCTGGAAGC-3’** |
| **Ta22845** | **TC353778** | **202** | **76.00** | **5’-GCTGGCTCGTTCAACTGATG-3’** | **5’-GGACCAAGCGTTCTGATTACTC-3’** |
| **Ta4045** | **TC350975, TC327386** | **185** | **74.90** | **5’-CCTGCCCCGTACAACCTTGAG-3’** | **5’-CACCGTTGCGATAGTCCTGAAAC-3’** |
| **Ta55512** | **TC311052, TC278654** | **173** | **76.90** | **5’-GCAAATCAGTGGAACAGAGG-3’** | **5’-ACAGCATCAGAGGACAAGG-3’** |
| **Ta53937** | **TC300803, TC279938** | **201** | **78.00** | **5’-CAAGATGATGAATGGCTAAATGG-3’** | **5’-AGGCACACCGTATGAACC-3’** |
| **Ta54227** | **TC308517, TC281050** | **227** | **80.40** | **5’-CAAATACGCCATCAGGGAGAACATC-3’** | **5’-CGCTGCCGAAACCACGAGAC-3’** |
| **Ta54733** | **TC335842, TC286574** | **243** | **78.20** | **5’-GACCACCTGCGTCTCCCTCAAA-3’** | **5’-ACAGATTCAGCCTTCCAGCCATTACC-3’** |
| **Ta53919** | **TC320440, TC279487** | **191** | **78.70** | **5’-GGCTGGACAAGAAGAAG-3’** | **5’-ATGGATGGTGGAGACG-3’** |
| **Ta1698** | **TC283369, TC282416** | **155** | **75.70** | **5’-GCAAGAACTATGACGGAGATG-3’** | **5’-CTGGTGAACACGGAAATGG-3’** |
| **Ta54448** | **TC279558** | **181** | **76.80** | **5’-TGCCATTCACAAATCAATC-3’** | **5’-AAACCCGACCTTAATCTTC-3’** |
| **Ta54512** | **TC284040, TC283036, TC284818** | **128** | **78.70** | **5’-AAGCAGCCGCACTACAAC-3’** | **5’-TAGACAGACTCCGCCTTGG-3’** |
| **Ta54447** | **TC279279, TC283613** | **202** | **78.90** | **5’-GAGTTTCGCTGCTGTTG-3’** | **5’-GGCTCCTCCATTCACG-3’** |
| **Ta53891** | **TC293469** | **102** | **76.20** | **5’-GTGGTGAAGGCGGAGAAG-3’** | **5’-GCACGATGACGAGGTAGC-3’** |
| **Ta53889** | **TC317382, TC333515** | **61** | **76.40** | **5’-CCGCCGCCAGGTCACAGG-3’** | **5’-GCTCGCCGCATCAAGGACAC-3’** |
| **Ta53967** | **TC292895, TC344391** | **235** | **80.80** | **5’-AGTGCTCGGTATCTACGG-3’** | **5’-TGCGAAGATGAGGATGAGG-3’** |
