# Supplementary figures and images for: Identification and validation of reference genes for quantitative RT-PCR normalization in wheat
Source: BMC Mol Biol. 2009 Feb 20;10:11. doi: 10.1186/1471-2199-10-11 (PMC2667184; doi:10.1186/1471-2199-10-11)

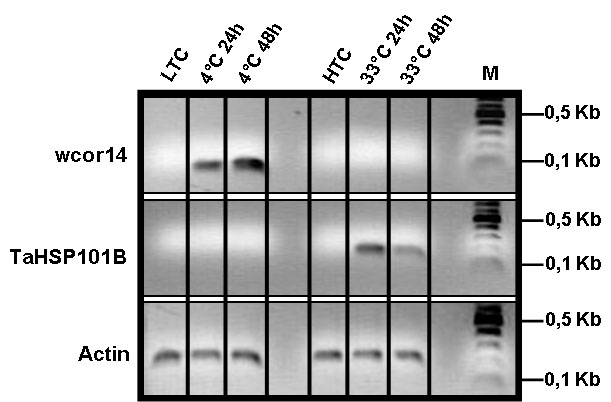

Supplement: Additional file 7 — Expression analysis by RT-PCR of the genes wcor14 and TaHSP101B. Agarose gels of RT-PCR products of wcor14 and TaHSP101B genes after 35 PCR cycles in six samples consisting of two temperature treatments (4°C and 33°C) for 24 and 48 h and their controls (LTC = low temperature controls; HTC = high temperature controls). The transcripts of the constitutively expressed gene encoding actin (UniGene cluster Ta54825) were amplified as control. M = part of the DNA molecular weight marker XIV (Roche), the most intense band is 500 bp in length. [file 1471-2199-10-11-S7.doc]

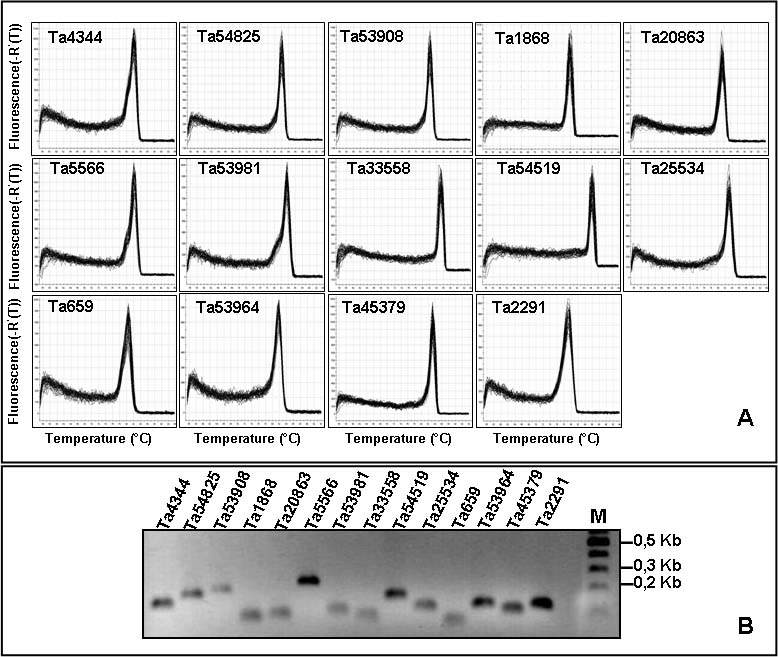


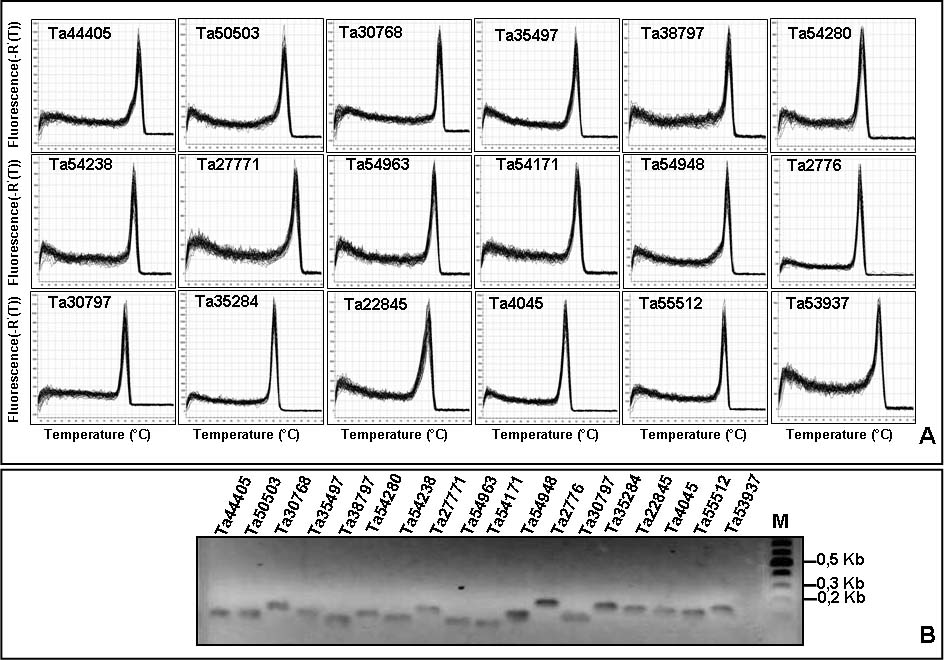


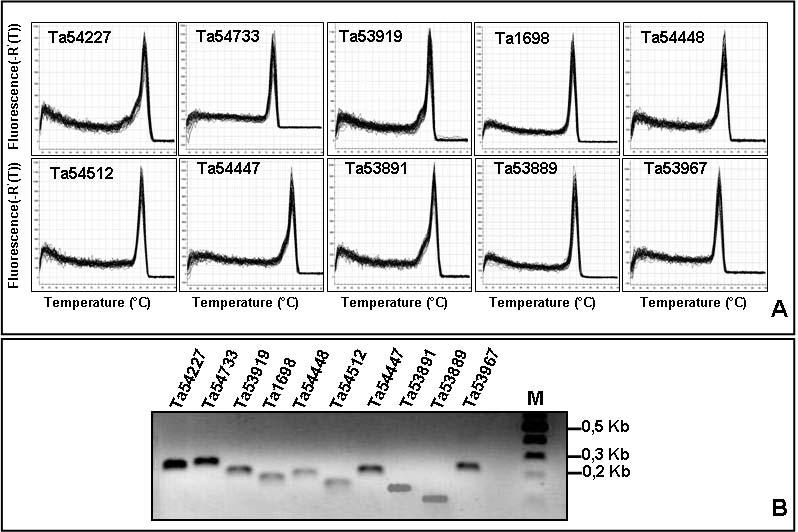

Supplement: Additional file 8 — Specificity of qRT-PCR amplification. Figures A1–A3 report the dissociation curves of 42 selected reference genes showing single peaks, they were obtained from three technical replicates of 18 cDNA pools representing different tissues and developmental stages of wheat. Figures B1–B3: Agarose gels (2%) showing amplification of a specific PCR product of the expected size for each candidate reference gene. [file 1471-2199-10-11-S8.doc]

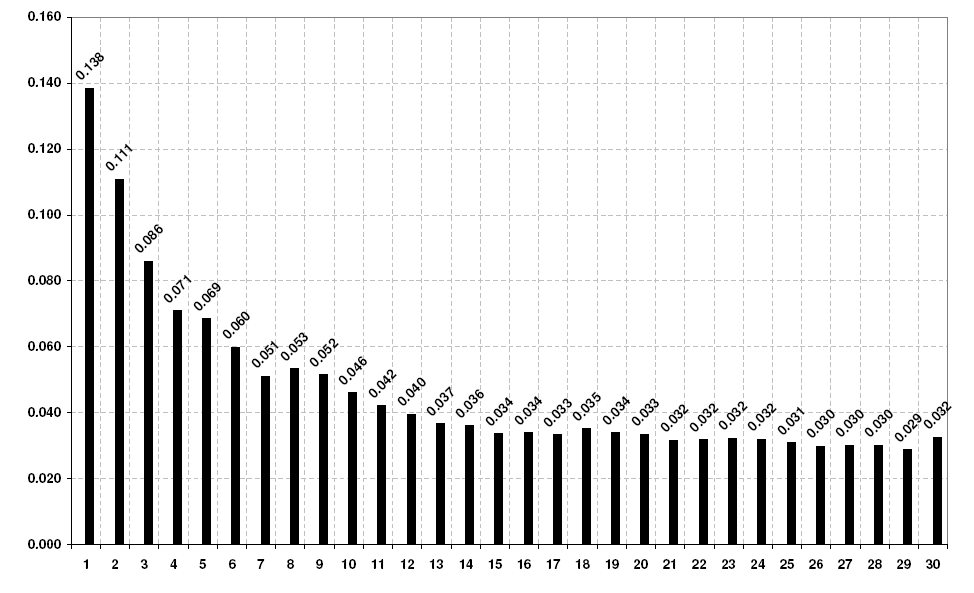


**A)**

**Pairwise variation (V)**


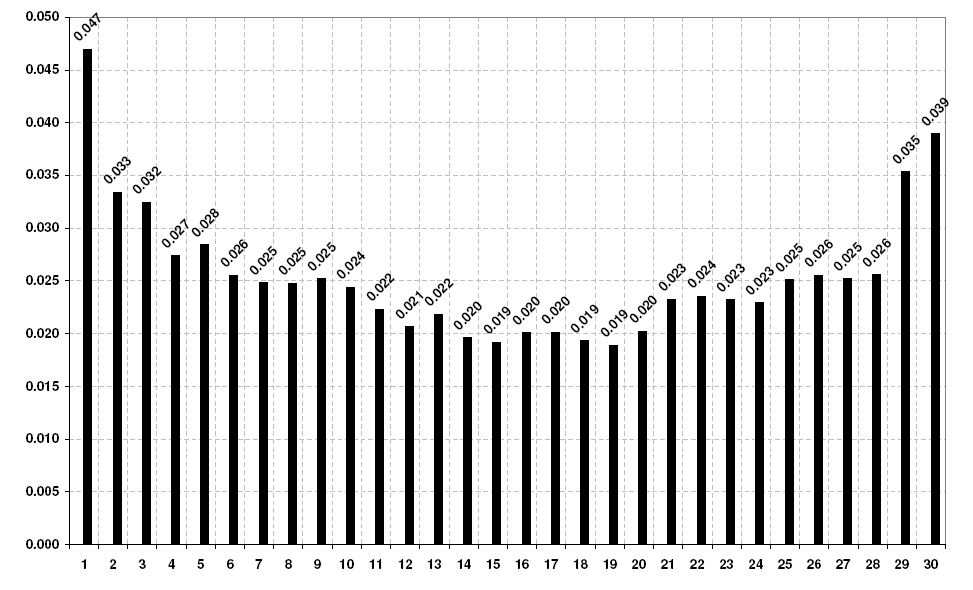


**B)**

**Pairwise variation (V)**


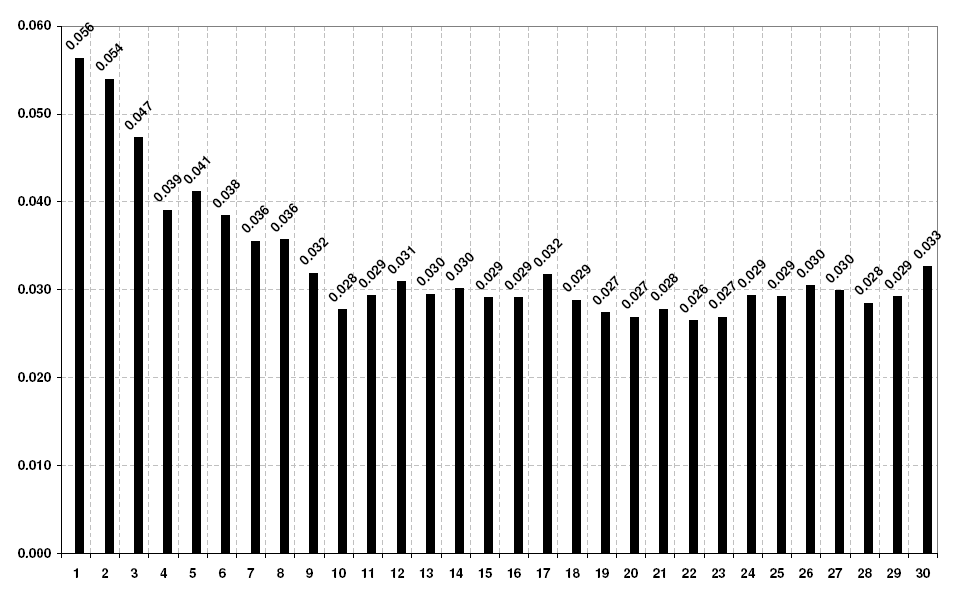


**C)**

**Pairwise variation (V)**


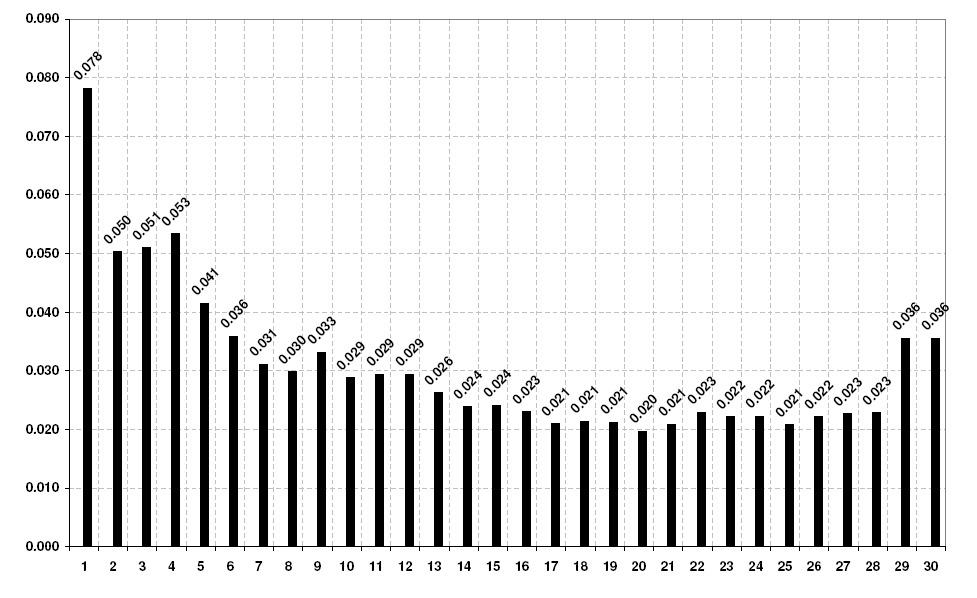


**D)**

**Pairwise variation (V)**

Supplement: Additional file 12 — Determination of the optimal number of control genes for accurate normalization calculated on the basis of pair-wise variation (V) analysis in four data sets. geNorm output charts of V values for the 32 selected reference genes in four data sets: (A) = 18 tissues and developmental stages; (B) = six samples consisting of two temperature treatments (4°C and 33°C) for 24 and 48 h and their controls; (C) = six floral organs from fully emerged spikes; (D) = six vegetative tissues and developmental stages (shoots, stems and leaves). [file 1471-2199-10-11-S12.doc]
