# Supplementary material for: Identification and validation of reference genes for quantitative RT-PCR normalization in wheat
Source: BMC Mol Biol. 2009 Feb 20;10:11. doi: 10.1186/1471-2199-10-11 (PMC2667184; doi:10.1186/1471-2199-10-11)
Supplement: Additional file 11 — Ranking of the expression stability of the reference genes as calculated by geNorm in four data sets. geNorm output charts of M values for the 32 selected reference genes in four data sets: (A) = 18 tissues and developmental stages; (B) = six samples consisting of two temperature treatments (4°C and 33°C) for 24 and 48 h and their controls; (C) = six floral organs from fully emerged spikes; (D) = six vegetative tissues and developmental stages (shoots, stems and leaves). [file 1471-2199-10-11-S11.doc]

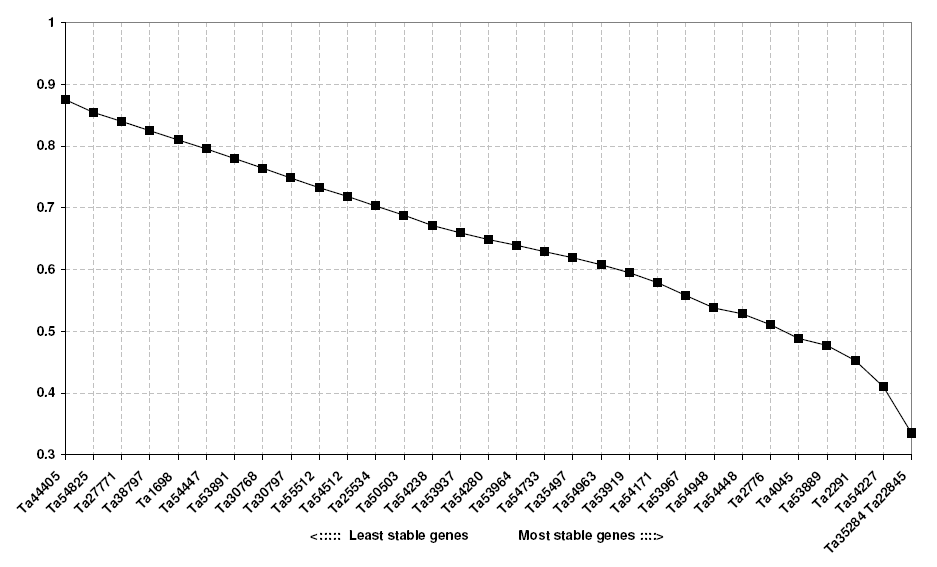


**A)**

**Average expression stability (M)**


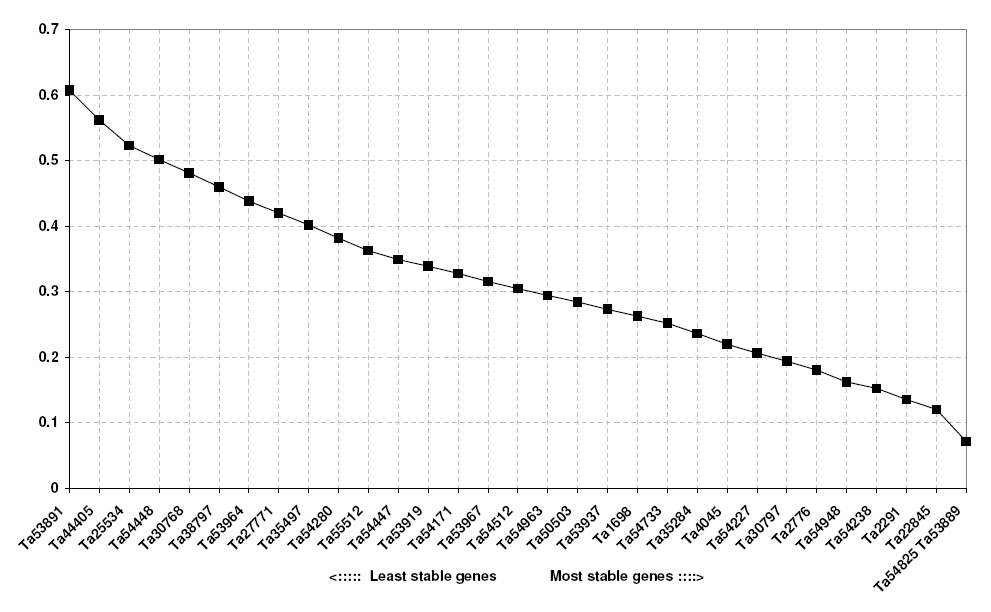


**B)**

**Average expression stability (M)**


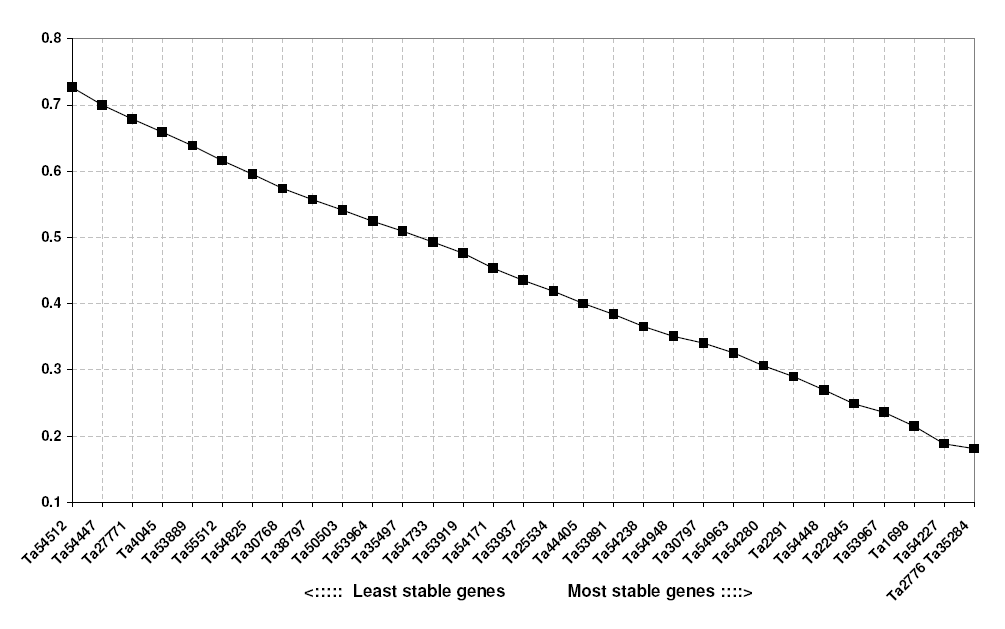


**C)**

**Average expression stability (M)**


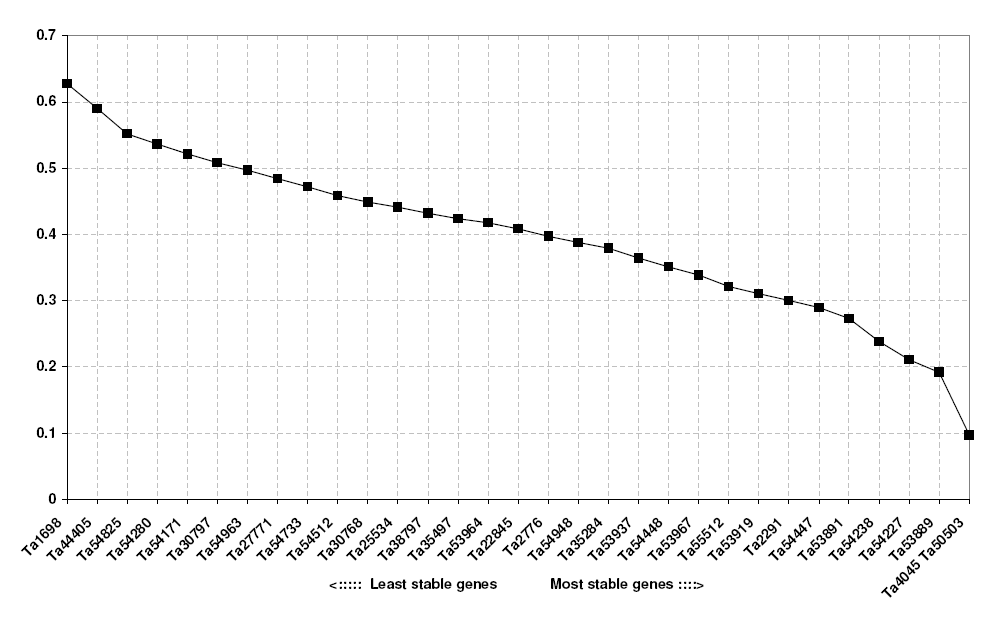


**D)**

**Average expression stability (M)**
